# Competing electrophilic substitution and oxidative polymerization of arylamines with selenium dioxide

**DOI:** 10.3762/bjoc.20.105

**Published:** 2024-05-27

**Authors:** Vishnu Selladurai, Selvakumar Karuthapandi

**Affiliations:** 1 Department of Chemistry, School of Advanced Sciences, VIT-AP University, Amaravati-522237, Andhra Pradesh, India

**Keywords:** arylamines, electrophilic substitution, oxamides: polymerization, selenium dioxide

## Abstract

This article describes the detailed analysis of the reaction between arylamines, such as aniline, *o*-anisidine, and methyl anthranilate, with selenium dioxide in acetonitrile. A systematic analysis of the reaction products with the help of ^77^Se NMR and single-crystal X-ray crystallography revealed that the reaction progress follows three major reaction pathways, electrophilic selenation, oxidative polymerization, and solvent oxidation. For aniline and *o*-anisidine, predominant oxidative polymerization occurred, leading to the formation of the respective polyaniline polymers as major products. For methyl anthranilate, the oxidative polymerization was suppressed due to the delocalization of amine lone pair electrons over the adjacent carboxylate function, which prompted the selenation pathway, leading to the formation of two of the isomeric diorganyl selenides of methyl anthranilate. The diaryl selenides were structurally characterized using single-crystal X-ray diffraction. Density functional theory calculations suggest that the highest occupied molecular orbital of methyl anthranilate was deeply buried, which suppressed the oxidative polymerization pathway. Due to solvent oxidation, oxamide formation was also noticed to a considerable extent. This study provides that utmost care must be exercised while using SeO_2_ as an electrophile source in aromatic electrophilic substitution reactions.

## Introduction

**Scheme 1:**
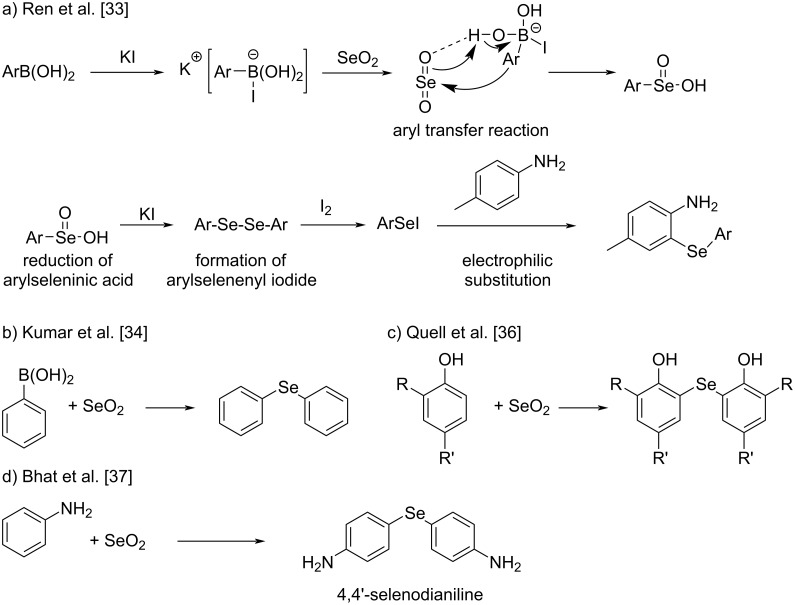
Reported synthetic methods for the selenation of aromatic compounds.

Organoselenium compounds have received considerable attention due to interesting medicinal properties, such as antioxidative [1–2], antimicrobial [3–4], and anticancer activity [5–6]. Several classes of organoselenium compounds are known to imitate the glutathione peroxidase [7–11]. Consequently, the development of new strategies for selenation of organic compounds has attracted considerable interest [12–13]. The various approaches used for selenation of aromatic compounds include directed lithiation [14–15], copper-catalyzed selenation [16–18], and aromatic nucleophilic substitution reactions [19–22]. Electrophilic selenium reagents (e.g., phenylselenenyl bromide) have often been used in oxyselenenylation of olefins, which follows an electrophilic addition mechanism [23–25]. However, such reagents are rarely used for electrophilic substitution of aromatic systems. Recently, notable progress has been made in the use of aromatic electrophilic substitution to synthesize arylselenium compounds [26–28]. Noteworthy examples are the use of SeO_2_ as selenium source in aromatic electrophilic substitution reactions [27–29]. Selenium dioxide is a well-known oxidizing agent for the allylic oxidation and oxidation of α-CH bonds located adjacent to electron-withdrawing groups [30–32]. Due to the oxidative nature, the use as selenium source in the synthesis of arylchalcogen compounds, in particular diorganyl selenides, can be a challenging process that involves sequential formation of two C–Se bonds and reduction of a Se=O bond in a single-pot reaction. Further, regioselective aromatic electrophilic substitution is often difficult. Various synthetic strategies have evolved to address such problems and expand the scope of SeO_2_ beyond the oxidizing capability. Ren et al. adopted potassium-iodide-mediated catalytic selenation of aromatic compounds using SeO_2_ (Scheme 1) [33]. This reaction comprises four main steps: (i) iodide-mediated aryl transfer from boronic acid to selenium dioxide, (ii) reduction of arylseleninic acid to diaryl diselenide, (iii) oxidation of diaryl diselenide to aryl selenenyl iodide with iodine, and (iv) electrophilic substitution of aniline derivatives. With this approach, electrophilic substitution can be achieved in *ortho* and *para* positions of aniline derivatives by careful selection of the substrates. Kumar et al. used SeO_2_ and phenylboronic acid to make symmetrical diaryl selenides [34]. In both cases, aryl transfer from the boron to the selenium center appeared to play a crucial role in the formation of the C–Se bond. AlCl_3_-catalyzed formation of benzoselenazole using SeO_2_ as electrophilic source has also been reported [35]. Quell et al. reported the syntheses of diaryl selenides and biphenol derivatives using SeO_2_ and phenols with one position (*ortho* or *para*) available for electrophilic substitution [36]. They found that solvent played a significant role in directing the outcome of the reaction. In protic solvents, biphenols were selectively formed through C–C bond formation, whereas in pyridine, the generation of diaryl selenide derivatives was almost exclusively promoted via aromatic electrophilic substitution. All of these reactions reveal the importance of using catalytic processes, preactivated substrates, or of blocking *ortho* or *para* sites to obtain the desired arylchalcogen compounds in good yield.

To our surprise, Bhat et al. have very recently reported the synthesis of the black solid 4,4'-selenodianiline without the use of catalysts, preactivation, or any blocking groups (Scheme 1) [37]. In contrast, Kim et al., utilizing a CuI-catalyzed reaction [38], have reported that 4,4'-selenodianiline is a pale brown solid, which conforms to our previous experience that diaryl selenides and diaryl diselenides are frequently yellow to brownish orange solids or liquids [39–40]. These contradicting observations prompted us to reinvestigate the reaction reported by Bhat et al. in detail. Indeed, we noticed that the reaction of SeO_2_ with arylamines follows a complex reaction pathway, leading to a mixture of compounds. We established the possible reaction pathways using ^77^Se NMR spectroscopy and single-crystal X-ray crystallographic studies. Density functional theory (DFT) calculations were carried out to understand the relative polymerization tendency of aniline, *o*-anisidine, and methyl anthranilate in the presence of SeO_2_.

## Results and Discussion

### Reaction of aniline with SeO_2_

**Scheme 2:**
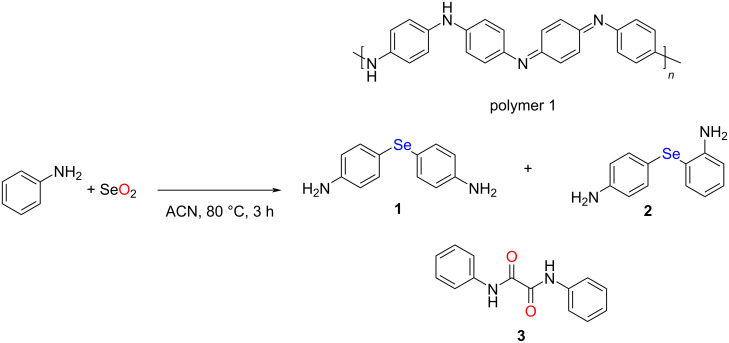
Reaction of selenium dioxide with aniline.

The reaction of aniline with SeO_2_ was carried out in acetonitrile in a nitrogen atmosphere (Scheme 2). It is worth mentioning that while the number of moles of reactants employed in our experiment was slightly different as compared to the condition mentioned in the work of Bhat. et. al [37], similar results were obtained under both conditions. Specifically, under our conditions, the amount of reactant used was 10 times higher than that used by Bhat. et. al., and the amount of solvent was doubled. We used a higher concentration of reactants in our studies as the yield of soluble products obtained at lower concentration of reactants was not satisfactory. However, under both conditions, a blackish purple polymeric solid was isolated as major product, labelled polymer 1. The polymer was isolated from the reaction after 3 h, and the supernatant was analyzed for other soluble products. The UV–vis spectrum of polymer 1 dispersed in methanol showed two major peaks at ≈271 and ≈561 nm (Figure S1, Supporting Information File 1), corresponding to the π→π* and benzenoid→quinonoid excitonic transitions, respectively, which was characteristic of polyaniline existing as emeraldine free base [41–42]. The polyaniline nature was further confirmed by FTIR spectroscopy (Figure S2, Supporting Information File 1). The broad peak observed in the range of 3300–3400 cm^−1^ was due to N–H stretching of the polymer [43–44]. The peak appearing at around 3000 cm^−1^ corresponded to the aryl C–H stretching. The peaks observed at 1593 and 1479 cm^−1^ were associated with C=C and C=N stretching, respectively. The obtained result was further supported by solid-state synthesis of selenious acid containing polyaniline via chemical oxidation of aniline with SeO_2_ as oxidant [45]. Further, Tanini et al. described that selenium dioxide has potential to oxidize aniline to form nitrobenzene in aqueous medium [46]. These findings irrevocably confirmed that aniline undergoes significant oxidative polymerization in the presence of SeO_2_.

After recognizing the polymeric nature of the solid, we analyzed the small molecular species present in the supernatant. Thin-layer chromatography (TLC) of the supernatant revealed that it contained a mixture of compounds. We could not isolate monoselenide **1** as a pure compound through column chromatography. The obtained fraction was a black solid as reported by Bhat et al., and HRMS analysis revealed a peak at *m*/*z* 265.0229, corresponding to [M + H]^+^ (Figure S4, Supporting Information File 1). However, we could not ascertain the identity of the compound based on HRMS data. To test whether the isolated black solid was a single compound or an isomeric mixture of compounds **1** and **2**, ^77^Se NMR spectroscopy could be a convenient tool. Unfortunately, ^77^Se NMR data was not reported in the work of Bhat et al. [37]. With this question in mind, the reaction was repeated several times to obtain a sufficient amount of the mixture for ^77^Se NMR spectroscopy. Indeed, two major resonance signals at 371 and 296 ppm were observed in the ^77^Se NMR spectrum of the mixture recorded in DMSO-*d*_6_ (Figure S5, Supporting Information File 1), which could be assigned to diaryl monoselenides **1** and **2**, respectively. The signal at 371 ppm corresponded to compound **1**, based on a previous report [47]. In turn, the other signal was assigned to the isomeric compound **2**. A similar observation was made in the ^77^Se NMR spectra of the pure diaryl diselenide isomers obtained from the reaction of methyl anthranilate with SeO_2_ (vide infra), which was also supported by single-crystal X-ray analysis. However, for aniline, in addition to the major polymerization and poor selenation process, solvent oxidation was also noted. Importantly, through column chromatography, the oxamide derivative **3** formed from through solvent (acetonitrile) oxidation was isolated as a colorless solid (vide infra). It was characterized by FTIR, ^1^H NMR, and mass spectrometry. The FTIR and mass spectrometry data were in accordance with the literature report [48–49]. Interestingly, the mass spectrum of the oxide **3** showed a signal at *m*/*z* 518.8590, corresponding to the 2:1 potassium complex [2M + K]^+^ (Figure S7, Supporting Information File 1).

### Reaction of *o*-anisidine with SeO_2_

**Scheme 3:**
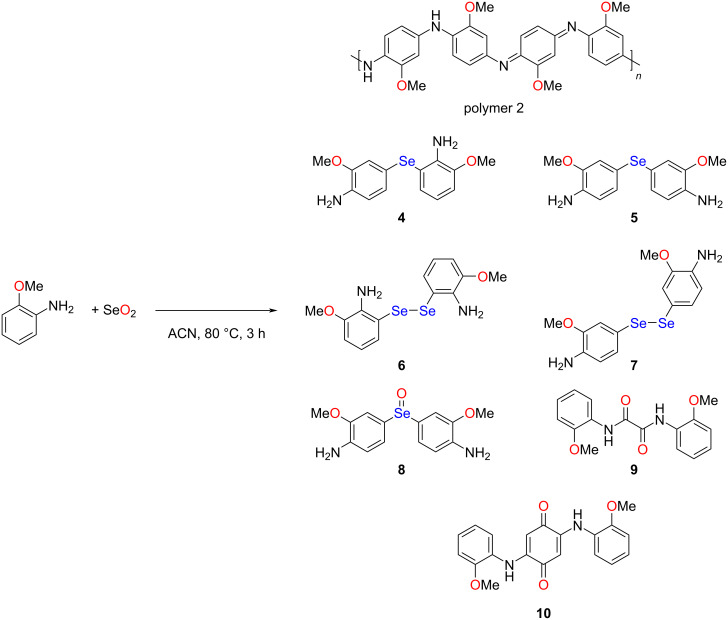
Reaction of selenium dioxide with *o*-anisidine.

To further understand the competitive process, we extended the reaction to *o*-anisidine with an electron-donating methoxy group in an *ortho* position relative to NH_2_. Scheme 3 shows the reaction of *o*-anisidine with SeO_2_. Similar to aniline, a large quantity of *o*-anisidine was transformed into polymer, labelled polymer 2. The UV–vis and FTIR spectra of polymer 2 are shown in Figures S1 and S2, Supporting Information File 1, respectively. The spectral characteristics were consistent with those of the emeraldine free base, as discussed above for polymer 1.

In the next step, the supernatant was analyzed by TLC and subsequently purified by column chromatography. Attempts to isolate the organoselenium compounds were unsuccessful due to the similar retention factor and low quantity. Therefore, all fractions were combined and subjected to ^77^Se NMR spectroscopy. The ^77^Se NMR spectrum of the mixture showed resonance signals at 406, 422, 537, 568, and 793 ppm (Figure S11, Supporting Information File 1). The peaks observed in the shielded region at 406 and 422 ppm were assigned to the unsymmetrical and symmetrical diaryl monoselenides **4** and **5**. But it was unclear which peak corresponded to which. Similarly, the peaks at 537 and 568 ppm could be tentatively assigned to diaryl diselenides **6** and **7**. The peak observed in the most deshielded region at 793 ppm was generally attributed of diaryl selenoxides such as compound **8** [39]. Despite the complexity, we could confirm the [M + H]^+^ ions of the isomeric monoselenides **4** and **5** from HRMS data (Figure S10, Supporting Information File 1). In addition to the above mentioned (possible) organoselenium compounds, the oxamide **9** and the quinone derivative 2,5-bis((2-methoxyphenyl)amino)cyclohexa-2,5-diene-1,4-dione (**10**) were isolated from the mixture. The spectroscopic data were consistent with earlier reports on the direct synthesis of **9** [50–51] and **10** [52]. The mass spectrum of oxamide **9** showed peaks corresponding to the sodium complexes [M + Na]^+^, [2M + Na]^+^, and [3M + Na]^+^ (Figure S13, Supporting Information File 1). The mass spectrum of quinone **10** showed a peak for [M + H]^+^ (Figure S17, Supporting Information File 1).

### Reaction of methyl anthranilate with SeO_2_

**Scheme 4:**
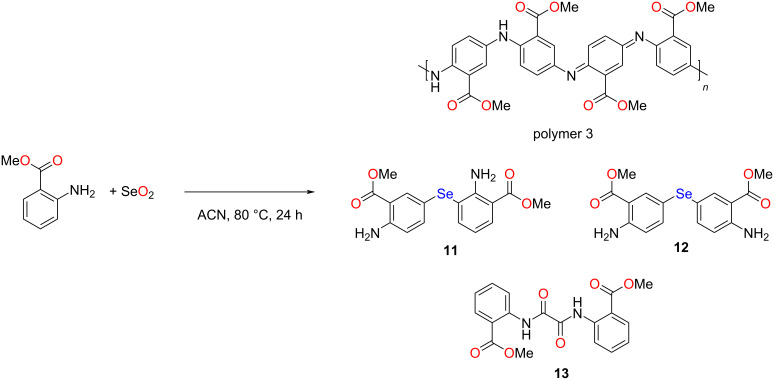
Reaction of methyl anthranilate with SeO_2_.

After observing that unsubstituted aniline and aniline with an electron-donating function (i.e., *o*-anisidine) undergo significant polymerization in the presence of SeO_2_, we were interested in studying this reaction using methyl anthranilate, having an electron-withdrawing group (Scheme 4). To our surprise, the quantity of polymer 3 formed was low. After isolating ≈360 mg of the semisolid blackish dark polymer 3, the supernatant was subjected to column chromatography, which afforded three compounds, namely diaryl monoselenides **11** (87 mg, 4.6%) and **12** (≈476 mg, 25%) as well as oxamide **13** (2.2 mg, 0.12%). Polymer 3 was characterized using UV and FTIR spectroscopy (Figures S1 and S2, Supporting Information File 1). The spectral data were consistent with the emeraldine free base. Further, a characteristic n→π* transition for the CO group was noticed in the UV spectrum of polymer 3, which was absent in the UV spectra of the other two polymers [53]. The mass spectra of purified compounds **11** and **12** showed peaks for [M + H]^+^ (Figures S21 and S26, Supporting Information File 1). The ^77^Se NMR spectra of the purified isomers **11** and **12** showed resonance signals at 300 and 394 ppm, respectively (Figures S24 and S29, Supporting Information File 1). Similar to the ^77^Se NMR signals of isomeric diaryl diselenides **1** and **2**, the symmetrical diaryl diselenide **12**, having an amino group *para* to the selenium center in both aryl units, showed a downfield shift compared to isomer **11**. The mass spectrum of oxamide **13** revealed peaks for the sodium complexes [M + Na]^+^, [2M + Na]^+^, and [3M + Na]^+^. Spectroscopic data of this compound, including FTIR as well as ^1^H and ^13^C NMR, matched those reported earlier [54].

### Relative extent of polymerization

To compare the relative polymerization tendency, we conducted reactions of arylamines with SeO_2_ in acetonitrile in sample vials and monitored the apparent color change (Figure S34, Supporting Information File 1). Instantaneous color change from colorless to blackish dark was observed for an *o*-anisidine solution after addition of SeO_2_. For an aniline solution, the color became light pink immediately upon addition of SeO_2_. For a methyl anthranilate solution, no color change was observed immediately upon addition of SeO_2_ as well as after 5 min of heating. The color of aniline and *o*-anisidine solutions became very intense after 5 min of heating. After 5 min of heating, the color intensity followed the following order: *o*-anisidine > aniline > methyl anthranilate. This clearly indicated that the relative extent of polymer formation decreased in the following order: *o*-anisidine > aniline > methyl anthranilate. As a consequence of the poor polymerization tendency, polymer 3 was obtained in a smaller amount of ≈360 mg after 24 h. Whereas at the same molar ratio, polymers 1 and 2 were obtained in larger quantities of ≈2.3 g after the short time of 3 h (see Experimental section for details).

### Mechanistic aspects

#### Mechanism for the formation of diaryl monoselenides

**Scheme 5:**
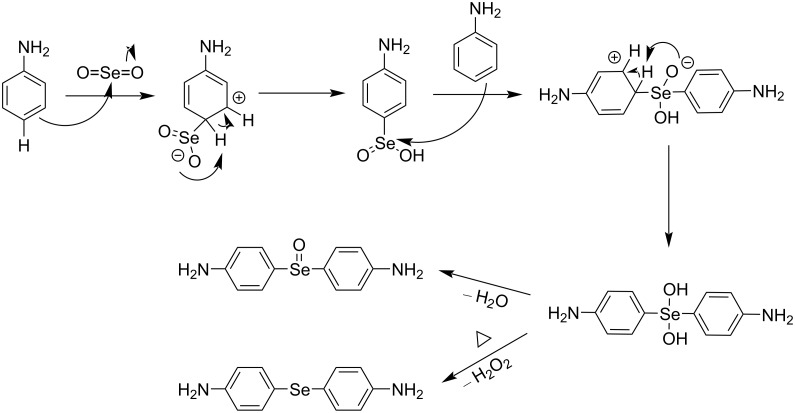
Reaction mechanism for the formation of diaryl monoselenides.

The plausible mechanism for the formation of diaryl monoselenides is shown in Scheme 5. The first step is the electrophilic substitution of SeO_2_ on the aromatic ring, either in the *ortho* or *para* position. The resulting arylseleninic acid acts as an electrophile, substituting a proton in another aniline molecule, leading to a hydrated selenoxide. This can give rise to either diaryl selenoxide via dehydration or diaryl monoselenide via reductive elimination by eliminating H_2_O_2_ [39]. Observation of *m*/*z* peaks for compound **8** clearly confirmed the formation of diaryl selenoxide in the reaction.

#### Mechanism for the formation of oxamides

**Scheme 6:**
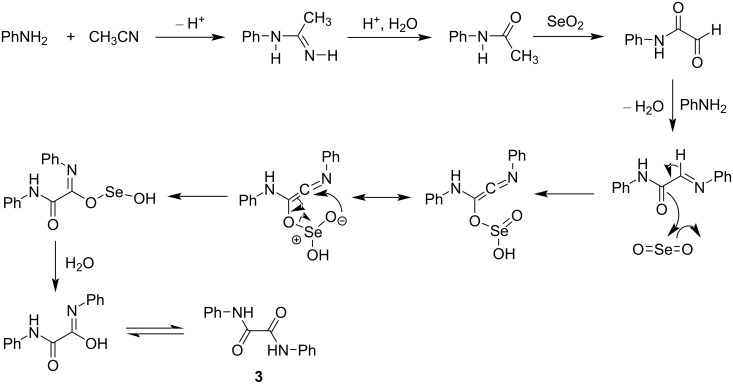
Reaction mechanism for the formation of oxamides.

The possible reaction mechanism for the formation of oxamide is shown in Scheme 6. Formation of acetanilide in the reaction of aniline and acetonitrile is known to occur in the presence of Lewis acid catalyst Al_2_O_3_ [55]. In our case, either SeO_2_ (Lewis acid) or H_2_SeO_3_ (Brønsted acid) may act as acid catalyst to convert aniline into acetanilide using acetonitrile as acetylating agent. The resulting acetanilide underwent α-CH oxidation with SeO_2_ to give 2-oxo-*N*-phenylacetamide, which undergoes Schiff base formation with aniline. The resulting Schiff base undergoes further α-CH oxidation with SeO_2_ to give the oxamide **3**. A similar mechanism may be assumed for the formation of the other oxamides **9** and **13**.

#### Mechanism for the formation of 2,5-bis((2-methoxyphenyl)amino)cyclohexa-2,5-diene-1,4-dione (**10**)

**Scheme 7:**
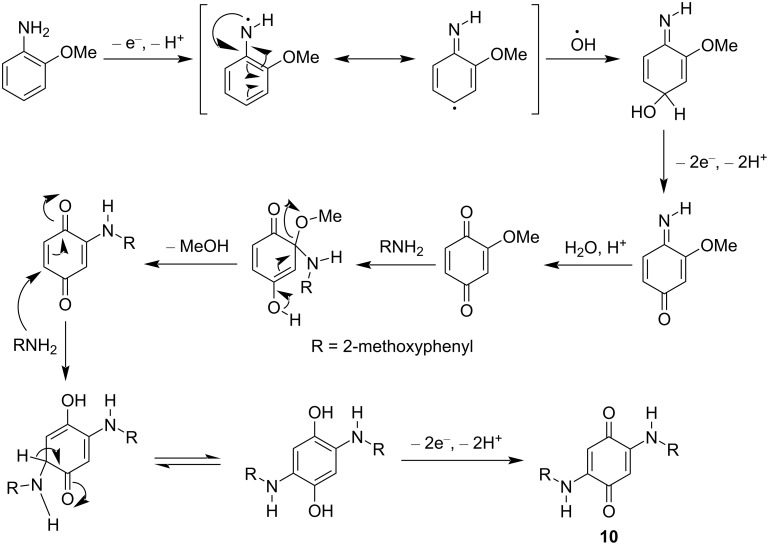
Reaction mechanism for the formation of quinone **10**.

Generally, formation of polyaniline occurs through a radical mechanism. Such a radical mechanism is relevant for the formation of quinones having exceptional radical-stabilizing abilities. The best example in nature is the radical pathway in the catechol oxidation process [56–58]. The structure of *o*-anisidine resembles catechol as it has two adjacent electron-donating functions (NH_2_ and OMe). For *o*-anisidine, the amine radical resulting from reaction of *o*-anisidine with SeO_2_ is stabilized by resonance (Scheme 7). It combines with the hydroxyl radical and undergoes subsequent oxidation to give 2-methoxycyclohexa-2,5-diene-1,4-dione. This iminoquinone upon hydrolysis gives 2-methoxycyclohexa-2,5-diene-1,4-dione, and the latter undergoes addition–elimination reaction with another molecule of *o*-anisidine to give 2-(phenylamino)cyclohexa-2,5-diene-1,4-dione. The third unit of *o*-anisidine is added to the quinone via 1,4-addition, followed by oxidation of the 1,4-dihydroxy compound to give 2,5-bis((2-methoxyphenyl)amino)cyclohexa-2,5-diene-1,4-dione (compound **10**).

### Single-crystal X-ray crystallographic studies

**Figure 1:**
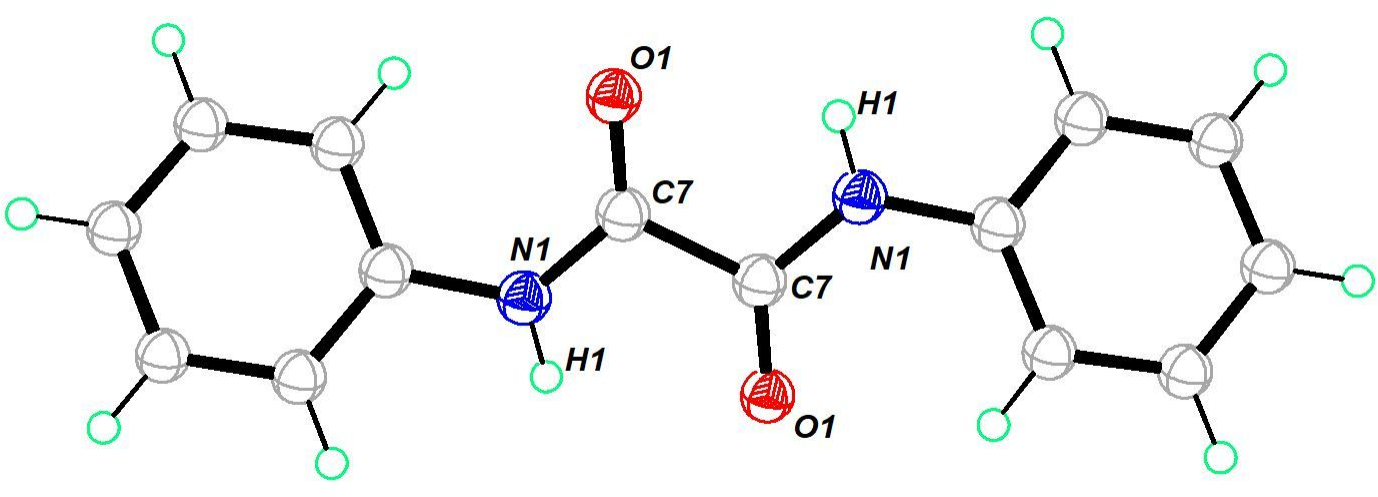
Molecular structure of **3**. Thermal ellipsoids drawn at 50% probability. Selected bond lengths (Å): O(1)–C(7) 1.2207 (16), N(1)–C(7) 1.3356 (17), N(1)–C(1) 1.4193 (16), N(1)–H(1) 0.8600.

The ORTEP diagram of oxamide **3** is shown in Figure 1. Compound **3** crystallized in a monoclinic crystal system in the *P*2_1_/*n* space group. The single-crystal X-ray structure of compound **3** was reported with the space group *P*2_1_/*c* [49]. It adopts a transoid geometry around the oxamide C–C bond with nearly 180° torsion angle. This provides the molecule with a planar geometry. It shows intermolecular hydrogen bonding between the amide O and NH moieties. (Figure S35, Supporting Information File 1).

**Figure 2:**
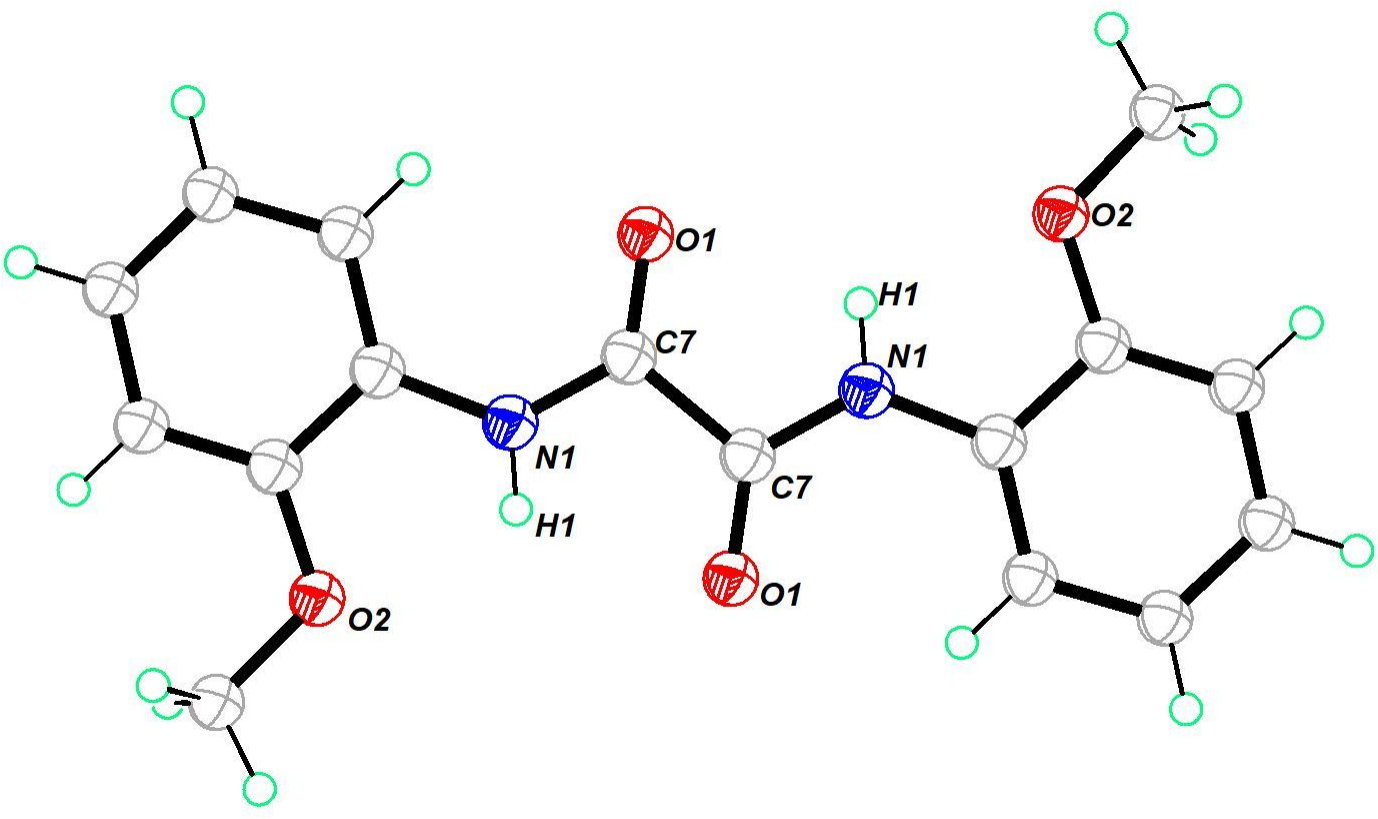
Molecular structure of **9**. Thermal ellipsoids drawn at 50% probability. Selected bond lengths (Å): O(1)–C(7) 1.2192 (18), N(1)–C(7) 1.3396, N(1)–C(1) 1.4050 (19), N(1)–H(1) 0.8600, O(2)–C(2) 1.3674 (18), O(2)–C(8) 1.420 (2).

Oxamide **9** crystallized in a monoclinic crystal system in the space group *P*2_1_/*c*. The structure was similar to the structure of oxamide **3** (Figure 2). The hydrogen of the NH unit was concealed by the oxygen atoms of the C=O and OMe units. Consequently, it did not engage in intermolecular hydrogen bonding. Due to the planar structure, it self-organized through π–π interaction between the oxamide motif and the aryl π-framework. Further, the CH^…^O=C interaction facilitated the formation of ladder-like packing in the solid state (Figure S36, Supporting Information File 1).

**Figure 3:**
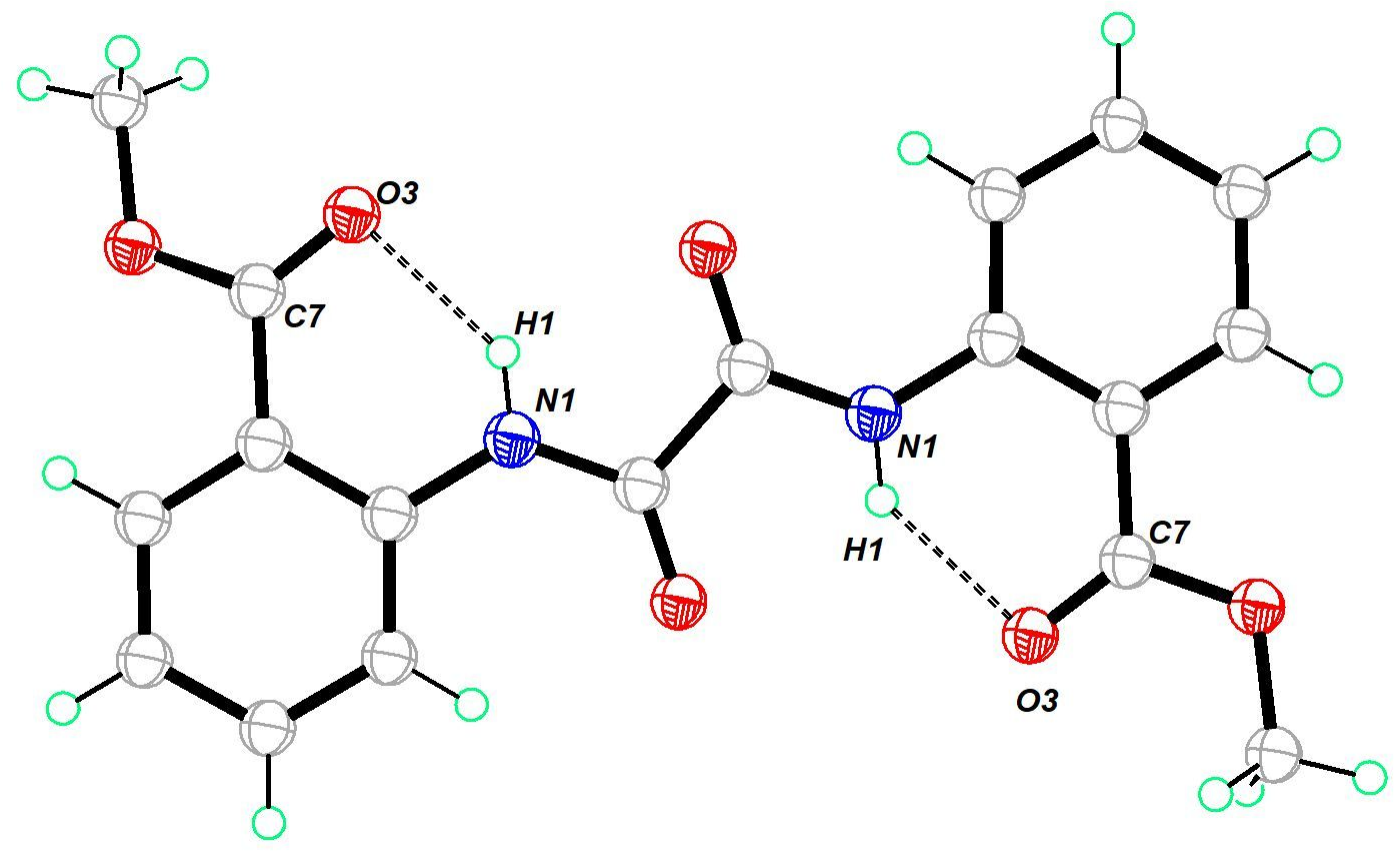
Molecular structure of **13**. Thermal ellipsoids drawn at 50% probability. Selected bond lengths (Å): O(3)–C(7) 1.2070 (16), O(1)–C(7) 1.3322 (16), N(1)–H(1) 0.8600, N(1)–C(6) 1.4044 (16), N(1)–C(9) 1.3440 (16), O(2)–C(9) 1.2160 (16).

The molecular structure of oxamide **13** is shown in Figure 3. It crystallized in a triclinic crystal system in the space group *P*−1. It was structurally similar to the other oxamides **3** and **9**. It showed an intramolecular hydrogen bonding between the NH and the carbonyl group of the methoxy ester. A layer-by-layer packing was observed in the crystal structure (Figure S37, Supporting Information File 1). The dipolar characteristics of the ester group appeared to assist the molecular layer-by-layer stacking seen in the crystal packing.

**Figure 4:**
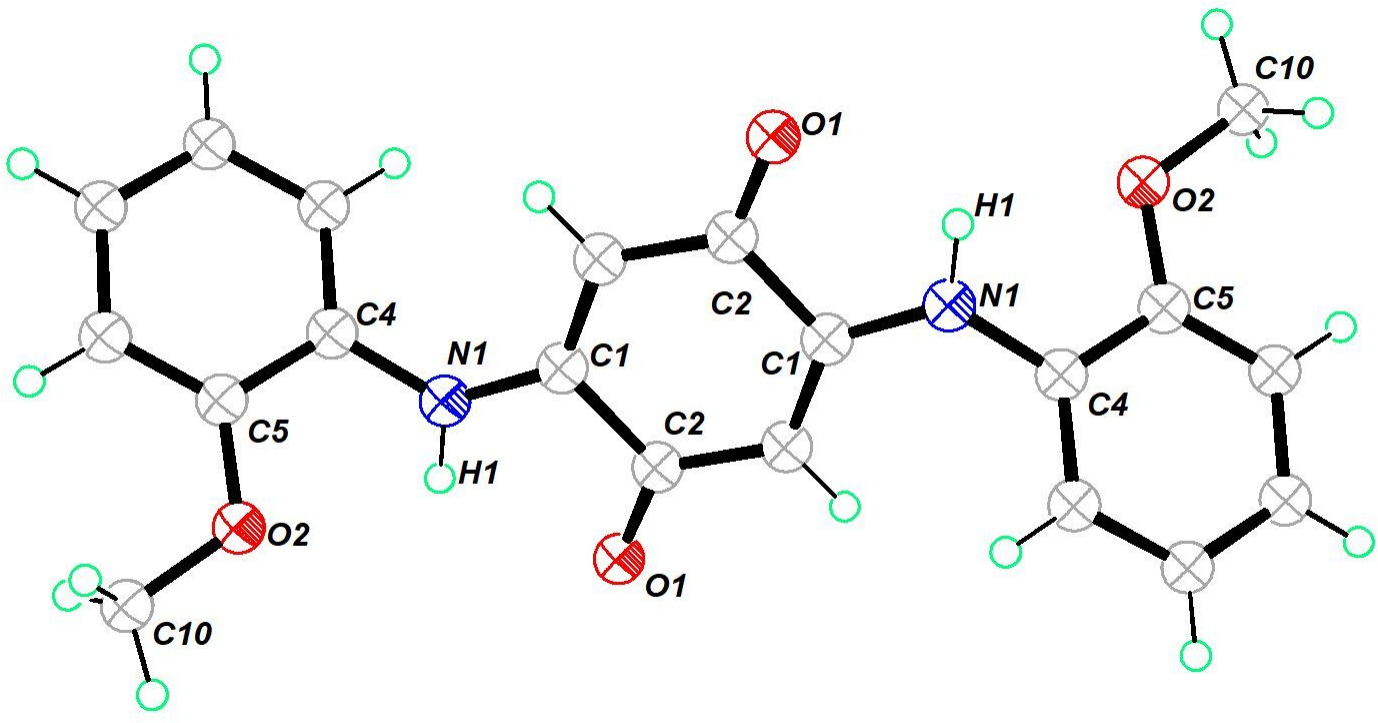
Molecular structure of **10**. Thermal ellipsoids drawn at 50% probability. Selected bond lengths (Å) and angles (°): O(1)–C(2) 1.2300 (16), N(1)–C(1) 1.3461 (17), N(1)–H(1) 0.884 (19), N(1)–C(4) 1.4078 (18), O(2)–C(5) 1.3610 (17), O(2)–C(10) 1.4308 (18), C(5)–O(2)–C(10) 118.06, C(1)–N(1)–C(4) 128.39 (12).

The molecular structure of the quinone derivative **10** is shown in Figure 4. It crystallized in a monoclinic crystal system in the space group *C*2/*c*.

**Figure 5:**
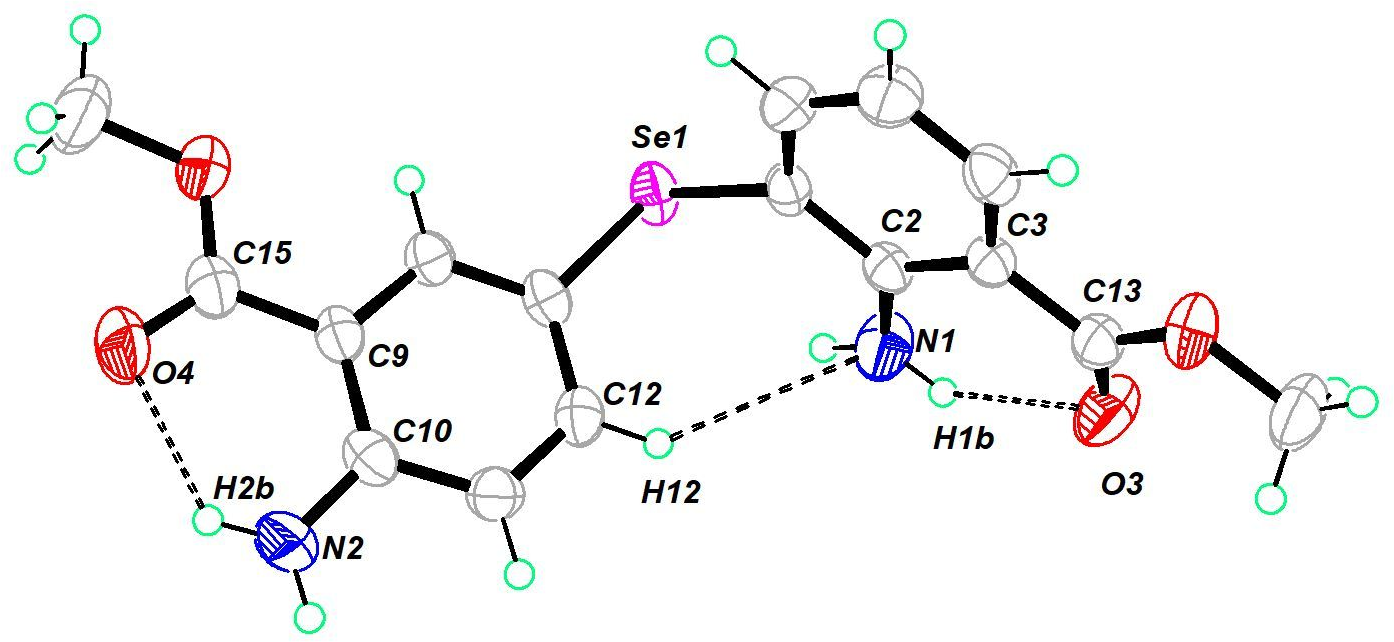
Molecular structure of **11**. Thermal ellipsoids drawn at 50% probability. Selected bond angles (°): C(7)–Se(1)–C(1) 99.01 (8), N(1)–C(2)–C(1) 120.34 (18), N(1)–C(2)–C(3) 122.05 (18), O(3)–C(13)–O(1) 121.5(2), O(3)–C(13)–C(3) 125.4 (2), N(2)–C(10)–C(9) 123.04 (19), O(4)–C(15)–O(2), O(4)–C(15)–O(2) 121.5 (2), O(4)–C(15)–C(9) 125.4 (2).

The molecular structure of the diorganyl monoselenide **11** is shown in Figure 5. It crystallized in a triclinic crystal system in the space group *P*−1. The C–Se–C bond angle was found to be 99.01°. Both inter- and intramolecular hydrogen bonding were noted in the structure (Figure S39, Supporting Information File 1). It allowed the molecule to self-align in a repeating cyclic ring pattern in one dimension.

**Figure 6:**
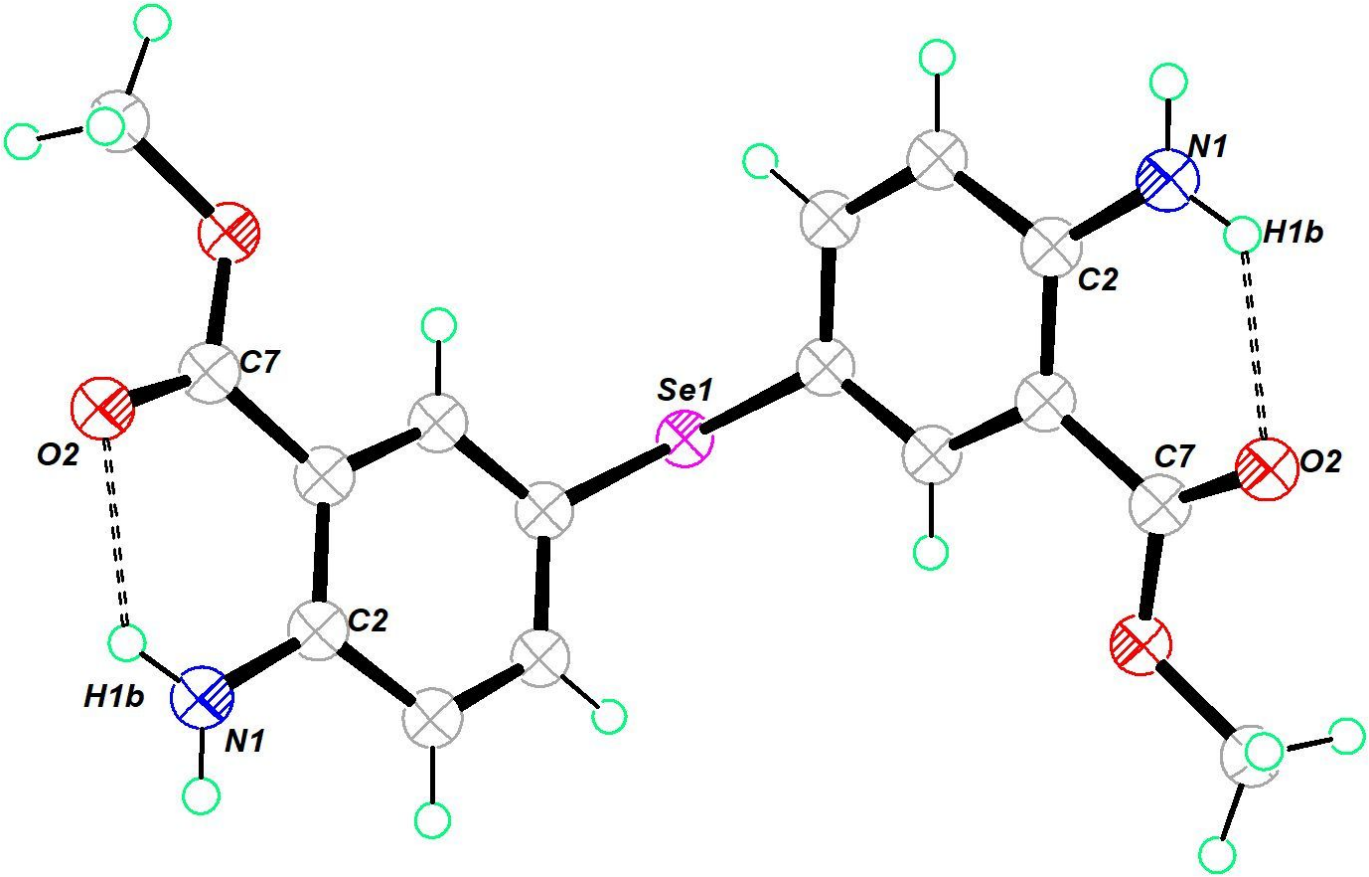
Molecular structure of **12**. Thermal ellipsoids drawn at 50% probability. Selected bond angles (°): C(5)–Se(1)–C(5) 100.11 (14) , C(7)–O(1)–C(8) 115.4 (2), O(2)–C(7)–O(1) 121.7 (2), O(2)–C(7)–C(1) 124.7 (2), N(1)–C(2)–C(1) 123.1 (2), N(1)–C(2)–C(3) 119.3 (2).

The molecular structure of the diorganyl monoselenide **12** is shown in Figure 6. It crystallized in an orthorhombic crystal system in the space group *P*2_1_2_1_2. The C–Se–C bond angle was 100.11°. Since the NH_2_ groups in this molecule were further away from each other compared to the monoselenide **11**, the hydrogen bonding arrangement led to a two-dimensional packing with a repeating zigzag pattern. Curiously, this compound showed an unusual Se···C σ-hole–π chalcogen bonding. Such interactions are rarely reported and currently gaining interest [59].

### Computational studies

**Figure 7:**
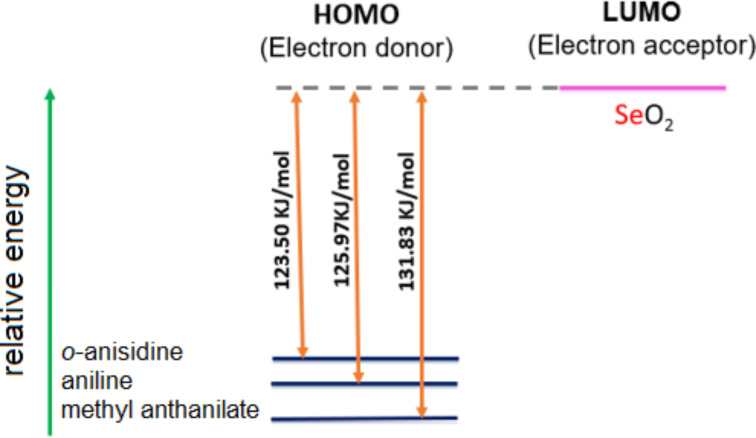
Relative energy levels of arylamines and SeO_2_.

To comprehend the role of electron-donating and -withdrawing groups in arylamine oxidation using SeO_2_ as oxidant, DFT calculations were carried out on the arylamines and SeO_2_ using Gaussian 16 [60]. The molecular structure of aniline, *o*-anisidine, and methyl anthranilate were optimized using the B3LYP/6-31G(d,p) basis set. Oxidative electron transfer from an arylamine to SeO_2_ was the first step in the oxidation process. This step was especially crucial for the polymerization of arylamines. Hence, we proposed that comparing the relative energy of the frontier molecular orbitals of arylamines with SeO_2_ could be a convenient measure to understand the differences in polymerization tendency. The relative energy of the frontier molecular orbitals of arylamines and SeO_2_ is shown in Figure 7. In an oxidative polymerization process, an electron is transferred from the HOMO of an arylamine donor to the LUMO of the SeO_2_ acceptor. The computed HOMO–LUMO energy difference (∆*E*) between arylamines and SeO_2_ decreased in the following order: *o*-anisidine − SeO_2_ > aniline − SeO_2_ > methyl anthranilate − SeO_2_. It revealed that the HOMO of methyl anthranilate was relatively buried, with a large energy gap (mismatch) between the donor and acceptor orbitals, which slowed down the oxidative polymerization process and allowed the alternative reaction pathway, electrophilic substitution of SeO_2_ on the aryl ring. This result was further supported by our observation (vide supra) that the polymerization of methyl anthranilate was extremely slow and yielded a lower quantity of polymer after 24 h reaction time.

**Figure 8:**
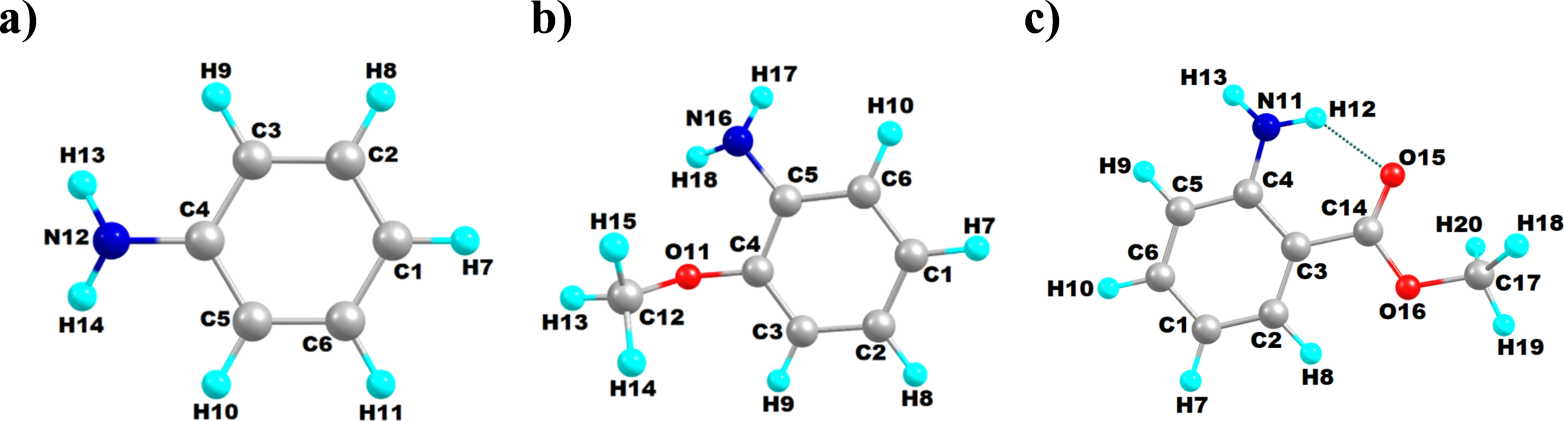
Computationally optimized structure of aniline (a), *o*-anisidine (b), and methyl anthranilate (c), with atom labels indicated.

**Scheme 8:**
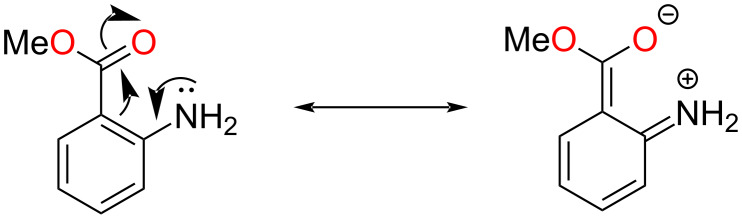
Resonance structures for the delocalization of the nitrogen lone pair into the π-system.

In the next step, natural bond orbital (NBO) analysis was carried out to understand the effect of electron delocalization on the arylamine reactivity. The NBO analysis was carried out using the same basis set, B3LYP/6-31G(d,p). The natural charge (*q*) of the nitrogen atom, occupancy of the nitrogen lone pair orbital, second-order perturbation energy (*E*) for intramolecular donor–acceptor interactions, and the donation of electron density form Lewis orbitals to non-Lewis orbitals obtained via NBO analysis of arylamines are provided in Table 1 [61]. The partial negative charge of the nitrogen atom was the lowest for methyl anthranilate when compared to aniline and *o*-anisidine. It was further correlated to the relatively low lone pair occupancy of methyl anthranilate, indicating poor electron density availability of the nitrogen atom for oxidation by SeO_2_. Therefore, the SeO_2_-mediated polymerization was slow for methyl anthranilate. The poor electron density of the nitrogen atom was due to the significant deviation of the methyl anthranilate structure from the ideal Lewis structure. The lone pair electron of nitrogen was donated to the non-Lewis orbital (i.e., LP*) of the adjacent carbon C(4) (see Figure 8), with a high interaction energy of 93.62 kcal⋅mol^−1^. Such an interaction was not found for aniline and *o*-anisidine. Instead, for *o*-anisidine and aniline, the lone pair electrons were delocalized into the antibonding NBO (i.e., BD*) with a smaller interaction energy of ≈26 kcal⋅mol^−1^. In other words, as shown in Scheme 8, the lone electron pair present at the nitrogen atom was delocalized into the π-conjugated system.

**Table 1:** Summary of NBO analysis.

arylamine	natural charge (*q*) of N	occupancy of N lone pair orbital	donor NBO	acceptor NBO	perturbation energy (*E*), kcal⋅mol^−1^

aniline	−0.83507	1.85302	LP–N12	BD*–C3–C4	25.99
*o*-anisidine	−0.84127	1.85122	LP–N16	BD*–C5–C6	25.64
methyl anthranilate	−0.81177	1.75677	LP–N11	LP*–C4	93.62

## Conclusion

Aniline and *o*-anisidine underwent a predominant oxidative polymerization reaction with SeO_2_. Therein, electrophilic selenation was poor. The reactivity of the NH_2_ unit towards oxidation could be partially suppressed by the presence of an electron-withdrawing function. Consequently, for methyl anthranilate, polymerization was suppressed, and selenated compounds were obtained with an appreciable yield. Further, when SeO_2_ was used as electrophile, solvent oxidation (e.g., interaction between solvent and acidic α-CH unit) and oxidation of reactive functions (e.g., NH_2_ group) had to be taken into account. Characterization of the organoselenium compounds by HRMS, ^1^H, and ^13^C NMR was supported by ^77^Se NMR and single-crystal X-ray analysis in order to confirm the identity of the compounds.

## Experimental

### General procedures

All syntheses were carried out using the standard Schlenk line in a nitrogen environment. Acetonitrile (99.9%) was bought from Avra Chemicals Private Ltd. and used without any further purification. Selenium dioxide and a range of reactants were purchased from Sigma-Aldrich. TLC was performed using silica-gel-coated aluminum sheets (TLC silica gel 60 F254). ^1^H, ^13^C, and ^77^Se NMR (500, 126, and 96 MHz) spectra were recorded in CDCl_3_ on a Bruker Avance III 500 MHz device. The chemical shift δ is reported in ppm, and the multiplicity of the signals is abbreviated as singlet (s), doublet (d), double of doublets (dd), triplet of doublets (td), etc. The coupling constants *J* are expressed in Hz. The calibration was done with respect to the signal of residual undeuterated solvent or in relationship to TMS (CDCl_3_: ^1^H 7.26 ppm, ^13^C 77.34 ppm). Single-crystal X–ray diffraction was recorded on a Bruker D8 Quest system. HRESIMS spectra were recorded on a Waters Xevo G2-XS QTof device. UV–vis spectra of all polymers were recorded on a PerkinElmer Lambda 365 UV–vis spectrophotometer. FTIR spectra were recorded on an Agilent Cary 630 KBr module.

### Reaction of aniline with SeO_2_

To a reaction vessel, 10 mL of acetonitrile were introduced along with aniline (10 mmol, ≈0.91 mL) and selenium dioxide (15 mmol, 1.66 g). The reaction mixture was stirred under a nitrogen atmosphere at 80 °C for 3 h. Before the solvent was evaporated at reduced pressure using a rotary evaporator, TLC was performed to verify completion of the reaction using 7:3 petroleum ether/ethyl acetate as the eluent. Upon evaporation of solvent, polymer 1 was obtained as a black solid. The solid was vacuum-filtered using a Büchner funnel and washed with acetonitrile. The solid was air-dried to give 2.33 g of a black crystalline solid that was insoluble in ethyl acetate and barely soluble in methanol. The filtrate was collected in a different round-bottom flask, and the solvent was evaporated in a rotary evaporator. The resulting solid was dissolved in DCM, and silica gel was added to the DCM layer. This slurry was subjected to column chromatography on silica gel (100–200 mesh) using petroleum ether and ethyl acetate as eluent. The initial pale yellow fraction was collected with 16% ethyl acetate, and the solvent was evaporated to give an off-white solid. Upon recrystallization from ethyl acetate, the above fraction afforded *N*^1^,*N*^2^-diphenyloxalamide (**3**) as a colorless solid. The remaining fractions were collected with 22% ethyl acetate. TLC suggested those fractions to be hardly separable. All fractions obtained with 20% ethyl acetate were combined and evaporated to give a black solid that contained a mixture of compounds, including the monoselenides **1** and **2**.

**Mixture of 4,4'-selenodianiline (1) and 2-((4-aminophenyl)selanyl)aniline (2):** Black solid (28.2 mg); ^77^Se NMR (95 MHz, DMSO-*d*_6_, δ) 371.2, 296.1; HRESIMS (*m*/*z*): [M + H]^+^ calcd for C_12_H_13_N_2_Se (*ortho*/*para*), 265.0238; found, 265.0229, [M + H − PhNH_2_]^+^ calcd for C_6_H_6_NSe (*ortho*/*para*), 172.0238; found, 171.9667; FTIR (KBr) ν̃_max_: 3436, 3354, 3220, 3026, 1595, 1490, 1282, 1073, 820, 752 cm^−1^.

***N*^1^,*N*^2^-Diphenyloxalamide (3):** Colorless solid (2.6 mg, 0.22%); mp 231–232 °C; ^1^H NMR (500 MHz, CDCl_3_, δ) 9.36 (s, 2H), 7.70 (d, *J* = 7.9 Hz, 4H), 7.44 (t, *J* = 7.8 Hz, 4H), 7.25 (t, *J* = 7.5 Hz, 2H); HRESIMS (*m*/*z*): [M + CH_3_COOH + H]^+^ calcd for C_16_H_17_N_2_O_4_, 301.1183; found, 301.1408, [2M + K]^+^ calcd for C_28_H_24_KN_4_O_4_, 519.1429; found, 518.8590; FTIR (KBr) ν̃_max_: 3354, 3063, 3019, 2840, 1684, 1602, 1528, 1252, 1021, 760 cm^−1^.

### Reaction of *o*-anisidine with SeO_2_

A round-bottom flask was charged with 10 mL of acetonitrile, *o*-anisidine (10 mmol, ≈1.13 mL), and selenium dioxide (15 mmol, ≈1.66 g). The flask content was stirred under a nitrogen atmosphere at 80 °C for 3 h. Evaporation of the reaction mixture on a rotary evaporator gave a black crystalline solid. The resulting solid was vacuum-filtered, washed with acetonitrile, and air-dried to yield 2.36 g of polymer 2 as a black solid. The filtrate was evaporated and column-chromatographed on silica gel (100–200 mesh) using petroleum ether and ethyl acetate as an eluent. The initial yellowish orange fraction containing *N*^1^,*N*^2^-bis(2-methoxyphenyl)oxalamide, collected with 22% ethyl acetate, was recrystallized from a chloroform/methanol mixture. The second reddish brown fraction containing 2,5-bis((2-methoxyphenyl)amino)cyclohexa-2,5-diene-1,4-dione was collected with 24% ethyl acetate. After evaporation of the solvent, the reddish brown precipitate was recrystallized from chloroform/methanol and ethyl acetate mixture as solvent. The fractions obtained with 32% ethyl acetate were mixtures of compounds. These fractions were combined and evaporated to afford a black semisolid containing **4**–**8**.

**Mixture of monoselenides 4 and 5, diselenides 6 and 7, and selenoxide 8:** Black solid (32 mg); ^77^Se NMR (95 MHz, CDCl_3_, δ) 792.52, 567.62, 537.42, 422.36, 405.91; HRESIMS (*m*/*z*): [M + H]^+^ calcd for C_14_H_17_N_2_O_2_Se (from **4** and **5**), 325.0450; found, 325.0497, [M + H]^+^ calcd for C_14_H_17_N_2_O_3_Se (from **8**), 341.0399; found, 341.0399, [M + H − PhNH_2_OMe]^+^ calcd for C_7_H_8_NOSe (from **4** and **5**), 201.9800; found, 201.1051; FTIR (KBr) ν̃_max_: 3436, 2922, 2855, 1617, 1580, 1498, 1222, 1028, 812, 745 cm^−1^.

***N*^1^,*N*^2^-Bis(2-methoxyphenyl)oxalamide (9)**: Yellow solid (4.2 mg, 0.29%); mp 220–221 °C; ^1^H NMR (500 MHz, CDCl_3_, δ) 9.96 (s, 2H), 8.42 (dd, *J* = 8.1, 1.7 Hz, 2H), 7.14 (td, *J* = 7.8, 1.6 Hz, 2H), 7.02 (td, *J* = 7.8, 1.4 Hz, 2H), 6.94 (dd, *J* = 8.2, 1.4 Hz, 2H), 3.94 (s, 6H); ^13^C NMR (126 MHz, CDCl_3_, δ) 157.5, 148.9, 126.2, 125.3, 121.0, 119.7, 110.3, 55.8; HRESIMS (*m*/*z*): [M + Na]^+^ calcd for C_16_H_16_N_2_NaO_4_, 323.1002; found, 323.1006, [2M + Na]^+^ calcd for C_32_H_32_N_4_NaO_8_, 623.2112; found, 623.2112, [3M + Na]^+^ calcd for C_48_H_48_N_6_NaO_12_, 923.3222; found, 923.3201; FTIR (KBr) ν̃_max_: 3354, 1684, 1602, 1528, 1461, 1252, 1021, 760 cm^−1^.

**2,5-Bis((2-methoxyphenyl)amino)cyclohexa-2,5-diene-1,4-dione (10):** Reddish brown solid (49 mg, 2.8%); mp 237–239 °C; ^1^H NMR (500 MHz, CDCl_3_, δ) 8.48 (s, 2H), 7.41 (dd, *J* = 7.9, 1.5 Hz, 2H), 7.16 (td, *J* = 7.9, 1.5 Hz, 2H), 7.04–6.93 (m, 4H), 6.17 (s, 2H), 3.91 (s, 6H); ^13^C NMR (126 MHz, CDCl_3_, δ) 180.6, 151.4, 145.6, 126.7, 125.9, 121.2, 120.8, 111.2, 96.4, 55.8; HRESIMS (*m*/*z*): [M + H]^+^ calcd for C_20_H_19_N_2_O_4_, 351.1339; found, 351.1350; FTIR (KBr) ν̃_max_: 3429, 3324, 3026, 2937, 1647, 1580, 1252, 1028, 738 cm^−1^.

### Reaction of methyl anthranilate with SeO_2_

To a round-bottom flask, 10 mL of acetonitrile were introduced along with methyl anthranilate (10 mmol, ≈1.29 mL) and selenium dioxide (15 mmol, ≈1.66 g). The reaction mixture was stirred under a nitrogen atmosphere at 80 °C for 24 h. The reaction progress was monitored using TLC. Evaporation of the reaction mixture on a rotary evaporator afforded polymer 3 as a semisolid along with other compounds. The semisolid was triturated with a 2:8 mixture of ethyl acetate and petroleum ether as solvent. Then, the supernatant was decanted into a beaker. The semisolid was allowed to dry, giving polymer 3 as black solid (0.36 g). The supernatant was concentrated, redissolved in DCM, and turned into a slurry by addition of silica gel (100–200 mesh) The slurry was subjected to column chromatography using petroleum ether and ethyl acetate as eluent. The first minor fraction was collected with 18% ethyl acetate. Upon evaporation of the solvent, dimethyl 2,2'-(oxalylbis(azanediyl))dibenzoate was obtained as a pale yellow solid and recrystallized from a 9:1 methanol/chloroform mixture. The second major fraction was collected with 22% ethyl acetate. Evaporation of the solvent afforded methyl 2-amino-3-((4-amino-3-(methoxycarbonyl)phenyl)selanyl)benzoate as pale yellow solid. It was recrystallized from a 9:1 methanol/chloroform mixture. The third major fraction was also collected with 22% ethyl acetate. After evaporation of the solvent, dimethyl 5,5'-selenobis(2-aminobenzoate) was obtained as yellow solid. The solid was recrystallized from a 9:1 methanol/chloroform mixture to afford a pale yellow solid.

**Methyl 2-amino-3-((4-amino-3-(methoxycarbonyl)phenyl)selanyl)benzoate** (**11**)**:** Pale yellow solid (87 mg, 4.6%); mp 152–153 °C; ^1^H NMR (500 MHz, CDCl_3_, δ) 8.02 (d, *J* = 2.2 Hz, 1H), 7.88 (dd, *J* = 8.1, 1.7 Hz, 1H), 7.68 (dd, *J* = 7.5, 1.7 Hz, 1H), 7.24 (dd, *J*=8.6, *J* = 2.1 Hz, 1H), 6.59–6.51 (m, 2H), 3.85 (s, 3H), 3.84 (s, 3H); ^13^C NMR (126 MHz, CDCl_3_) 168.5, 167.9, 150.9, 150.0, 142.2, 138.2, 135.5, 132.6, 118.0, 117.3, 116.0, 114.7, 111.4, 110.8, 51.7; ^77^Se NMR (95 MHz, CDCl_3_, δ) 300.2. HRESIMS (*m*/*z*): [M + H]^+^ calcd for C_16_H_17_N_2_O_4_Se, 381.0348; found, 381.0359, [M + H − PhNH_2_CO_2_Me]^+^ calcd for C_8_H_8_NO_2_Se, 229.9715; found, 229.9721; FTIR (KBr) ν̃_max_: 3213, 3078, 1699, 1580, 1505, 1267, 820 cm^−1^.

**Dimethyl 5,5'-selenobis(2-aminobenzoate) (12**)**:** Yellow solid (476 mg, 25.1%); mp 163–164 °C; ^1^H NMR (500 MHz, CDCl_3_, δ) 8.06 (d, *J* = 2.1 Hz, 2H), 7.35 (dd, *J* = 8.6, 2.1 Hz, 2H), 6.55 (d, *J* = 8.5 Hz, 2H), 5.77 (s, 4H), 3.84 (s, 6H); ^13^C NMR (126 MHz, CDCl_3_, δ) 168.0, 145.0, 139.5, 136.5, 117.8, 116.8, 111.3, 51.7; ^77^Se NMR (95 MHz, CDCl_3_, δ) 394.1; HRESIMS (*m*/*z*): [M + H]^+^ calcd for C_16_H_17_N_2_O_4_Se, 381.0348; found, 381.0356; FTIR (KBr) ν̃_max_: 3466, 3377, 1677, 1617, 1413, 1237, 1095, 752 cm^−1^.

**Dimethyl 2,2'-(oxalylbis(azanediyl))dibenzoate (13**)**:** Pale yellow solid (1.9 mg, 0.12%); mp 251–252 °C; ^1^H NMR (500 MHz, CDCl_3_, δ) 12.89 (s, 2H), 8.88 (dd, *J* = 8.4, 1.2 Hz, 2H), 8.11 (dd, *J* = 7.9, 1.6 Hz, 2H), 7.61 (ddd, *J* = 8.7, 7.3, 1.7 Hz, 2H), 7.24–7.17 (m, 2H), 4.01 (s, 6H); ^13^C NMR (126 MHz, CDCl_3_, δ) 168.1, 158.4, 139.6, 134.6, 131.2, 124.0, 120.5, 116.6, 52.7; HRESIMS (*m*/*z*): [M + Na]^+^ calcd for C_18_H_16_N_2_NaO_6_, 379.0901; found, 379.0924, [2M + Na]^+^ calcd for C_36_H_32_N_4_NaO_12_, 735.1909; found, 735.1898, [3M + Na]^+^ calcd for C_54_H_48_N_6_NaO_18_, 1091.2917; found, 1091.2885; FTIR (KBr) ν̃_max_: 3444, 3354, 1677, 1625, 1423, 1304, 1237, 820 cm^−1^.

### X-ray data

Table 2 and Table 3 show single-crystal X-ray structure refinement data.

**Table 2:** Single-crystal X-ray structure refinement data for **3**, **9**, and **10**.

parameter	**3**	**9**	**10**

empirical formula	C_14_H_12_N_2_O_2_	C_16_H_16_N_2_O_4_	C_20_H_18_N_2_O_4_
formula weight	240.26	300.31	350.36
crystal system	monoclinic	monoclinic	monoclinic
space group	*P*2_1_/*n*	*P*2_1_/*c*	*C*2/*c*
*a*, Å	5.3310(9)	7.7817(10)	19.843(4)
*b*, Å	5.3762(9)	14.954(2)	3.8540(8)
*c*, Å	20.182(3)	6.9263(9)	21.797(5)
α, °	90	90	90
β, °	93.598(5)	114.369(4)	96.420(8)
γ, °	90	90	90
*V*, Å^3^	577.29(17)	734.19(17)	1656.5 (6)
*Z*	2	2	4
ρ calcd, mg⋅m^−3^	1.382	1.358	1.405
absorption coefficient, mm^−1^	0.094	0.099	0.099
reflections collected	14639	16216	16841
final *R*(*F*) [*I* > 2σ(*I)*]	0.0570	0.0866	0.0615
*wR*(*F*^2^) indices [*I* > 2σ(*I*)]	0.1209	0.1303	0.1249
data/restraints/parameters	1410/0/82	1838/0/101	2061/0/122
goodness-of-fit on *F*^2^	1.072	1.051	1.068

**Table 3:** Single-crystal X-ray structure refinement data for **11**, **12**, and **13**.

parameter	**11**	**12**	**13**

empirical formula	C_16_H_16_N_2_O_4_Se	C_16_H_16_N_2_O_4_Se	C_18_H_16_N_2_O_6_
formula weight	379.27	379.27	356.33
crystal system	triclinic	orthorhombic	triclinic
space group	*P*−1	*P*2_1_2_1_2	*P*−1
*a*, Å	5.2732(10)	9.2080(17)	5.4243(6)
*b*, Å	11.353(2)	19.174(3)	8.3905(10)
*c*, Å	14.331(3)	4.5051(7)	9.2803(11)
α, °	105.300(6)	90	84.202(4)
β, °	97.375(6)	90	84.144(4)
γ, °	101.622(6)	90	75.208(4)
*V*, Å^3^	795.5(3)	795.4 (2)	405.01(8)
*Z*	2	2	1
ρ calcd, mg⋅m^−3^	1.583	1.584	1.461
absorption coefficient, mm^−1^	2.382	2.382	0.111
reflections collected	20734	31206	14437
final *R*(*F*) [*I* > 2σ(*I)*]	0.0352	0.0246	0.0438
*wR*(*F*^2^) indices [*I* > 2σ(*I*)]	0.0726	0.0586	0.1029
data/restraints/parameters	3595/0/210	1638/0/106	1646/0/119
goodness-of-fit on *F*^2^	1.035	1.095	1.039

## Supporting Information

File 1 Spectroscopic characterization of products (^1^H, ^13^C and ^77^Se NMR, IR, and HRMS spectra), packing arrangements of compounds and HOMO and LUMO energy values for reactants.

File 2 Crystallographic data for compound **3**.

File 3 Crystallographic data for compound **9**.

File 4 Crystallographic data for compound **10**.

File 5 Crystallographic data for compound **11**.

File 6 Crystallographic data for compound **12**.

File 7 Crystallographic data for compound **13**.

## Data Availability

All data that supports the findings of this study is available in the published article and/or the supporting information to this article.
