# Supplementary material for: Competing electrophilic substitution and oxidative polymerization of arylamines with selenium dioxide
Source: Beilstein J Org Chem. 2024 May 27;20:1221–35. doi: 10.3762/bjoc.20.105 (PMC11181186; doi:10.3762/bjoc.20.105)
Supplement: File 1 — Spectroscopic characterization of products (1H, 13C and 77Se NMR, IR, and HRMS spectra), packing arrangements of compounds and HOMO and LUMO energy values for reactants. [file Beilstein_J_Org_Chem-20-1221-s001.pdf]

BEILSTEIN JOURNAL OF ORGANIC CHEMISTRY

Supporting Information

for

Competing electrophilic substitution and oxidative polymerization of arylamines with selenium dioxide

Vishnu Selladurai and Selvakumar Karuthapandi

Beilstein J. Org. Chem. **2024,** *20,* 1221–1235. doi:10.3762/bjoc.20.105

Spectroscopic characterization of products (^{1}H, ^{13}C and ^{77}Se NMR, IR, and HRMS spectra), packing arrangements of compounds and HOMO and LUMO energy values for reactants

License and Terms: This is a supporting information file under the terms of the Creative Commons Attribution License (https://creativecommons.org/licenses/by/4.0). Please note that the reuse, redistribution and reproduction in particular requires that the author(s) and source are credited and that individual graphics may be subject to special legal provisions.
The license is subject to the *Beilstein Journal of Organic Chemistry* terms and conditions: (https://www.beilstein-journals.org/bjoc/terms)

Content page

1. Spectroscopic characterization of polymers 1–3 S3

UV–vis spectrum for polymers 1–3 S3
FTIR spectrum for polymers 1–3 S4

2. Spectrometric characterization of products obtained from the reaction of aniline with SeO_2 S5

FTIR spectrum for mixture of compounds **1** and **2** S5
HRMS–ESI spectrum for mixture of compounds **1** and **2** S6
^{77}Se NMR spectral studies for mixture of compounds **1** and **2** S7
FTIR spectrum for compound **3** S8
HRMS–ESI spectrum for compound **3** S9
^{1}H NMR spectral studies for compound **3** S10

3. Spectrometric characterization of products obtained from the reaction of *o*-anisidine with SeO_2 S11

FTIR spectrum for mixture of compounds **4**–**8** S11
HRMS–ESI spectrum for mixture of compounds **4**–**8** S12
^{77}Se NMR spectral studies for mixture of compounds **4**–**8** S13
FTIR spectrum for compound **9** S14
HRMS–ESI spectrum for compound **9** S15
^{1}H NMR spectral studies for compound **9** S16
^{13}C NMR spectral studies for compound **9** S17
FTIR spectrum for compound **10** S18
HRMS–ESI spectrum for compound **10** S19
^{1}H NMR spectral studies for compound **10** S20
^{13}C NMR spectral studies for compound **10** S21

4. Spectrometric characterization of products obtained from the reaction of methyl anthranilate with SeO_2 S22

FTIR spectrum for compound **11** S22
HRMS–ESI spectrum for compound **11** S23
^{1}H NMR spectral studies for compound **11** S24
^{13}C NMR spectral studies for compound **11** S25
^{77}Se NMR spectral studies for compound **11** S26
FTIR spectrum for compound **12** S27
HRMS–ESI spectrum for compound **12** S28
^{1}H NMR spectral studies for compound **12** S29
^{13}C NMR spectral studies for compound **12** S30
^{77}Se NMR spectral studies for compound **12** S31
FTIR spectrum for compound **13** S32
HRMS–ESI spectrum for compound **13** S33
^{1}H NMR spectral studies for compound **13** S34
^{13}C NMR spectral studies for compound **13** S35

5. Relative color change upon addition of SeO_2 to arylamines in acetonitrile S36

Photo images of reaction vessels S36

6. Packing arrangement for compounds **3** and **9** S37
7. Packing arrangement for compounds **13** and **10** S38
8. Packing arrangement for compound **11** S39
9. Packing arrangement for compound **12** S40

10. HOMO and LUMO energy values for reactants S41

1. **Spectroscopic characterization of polymers 1–3**

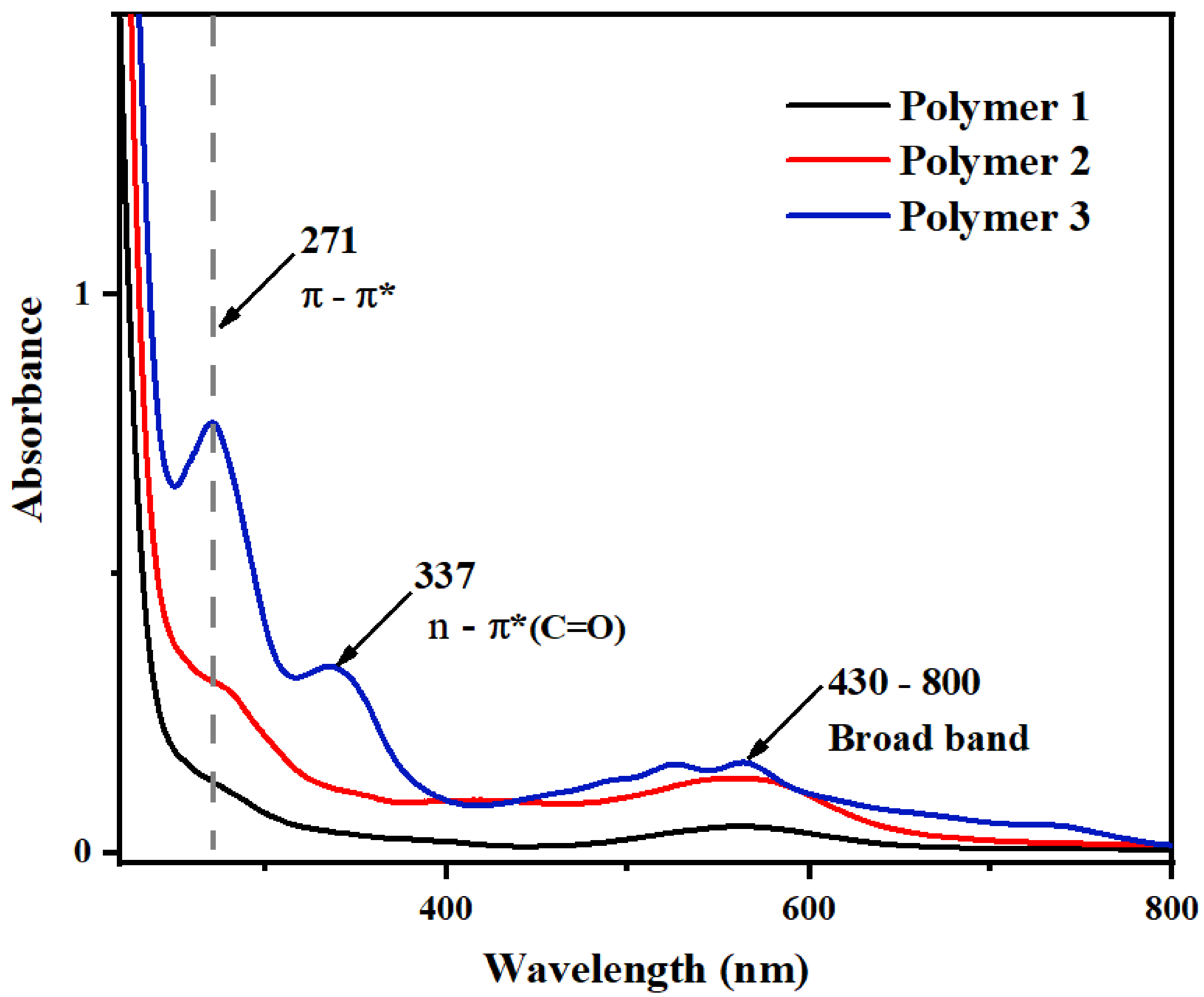

Figure S1. UV–vis spectrum for polymers 1–3.

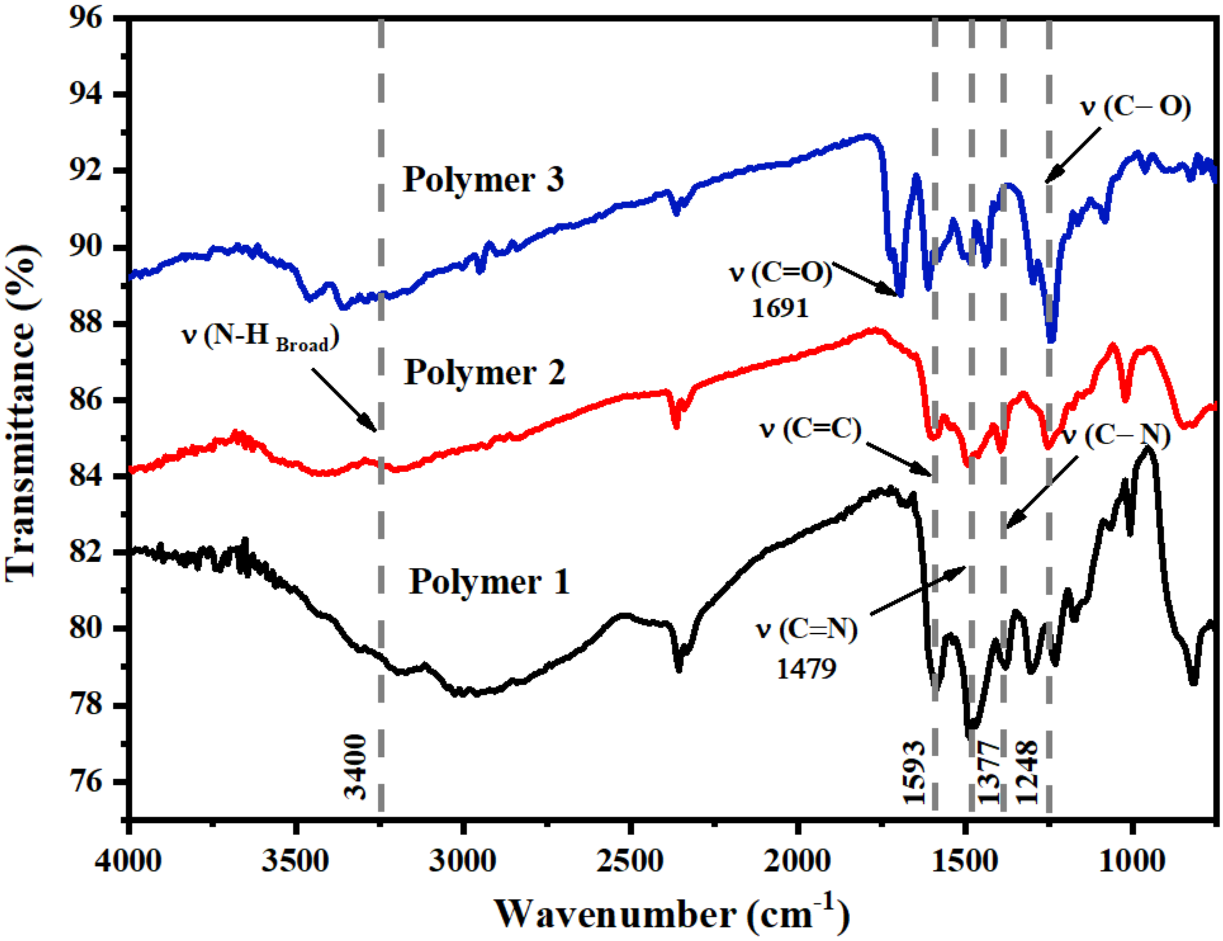

Figure S2. FTIR (KBr) spectrum for polymers 1–3.

2. Spectrometric characterization of products obtained for the reaction of aniline with SeO_2

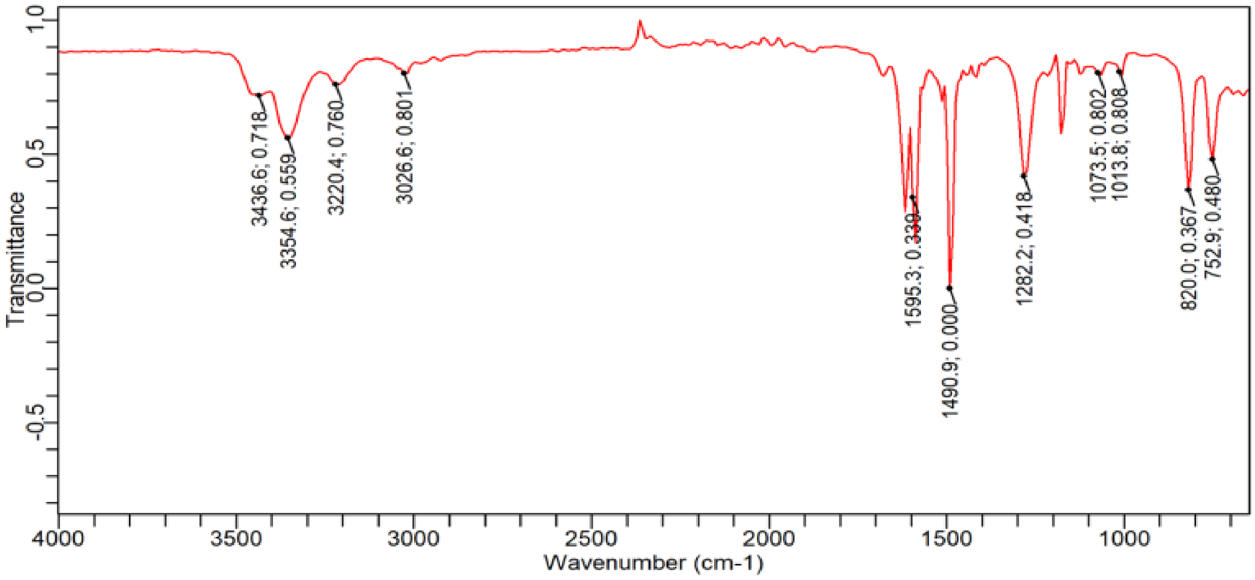

Figure S3. FTIR(KBr) spectrum for mixture of compounds **1** and **2**.

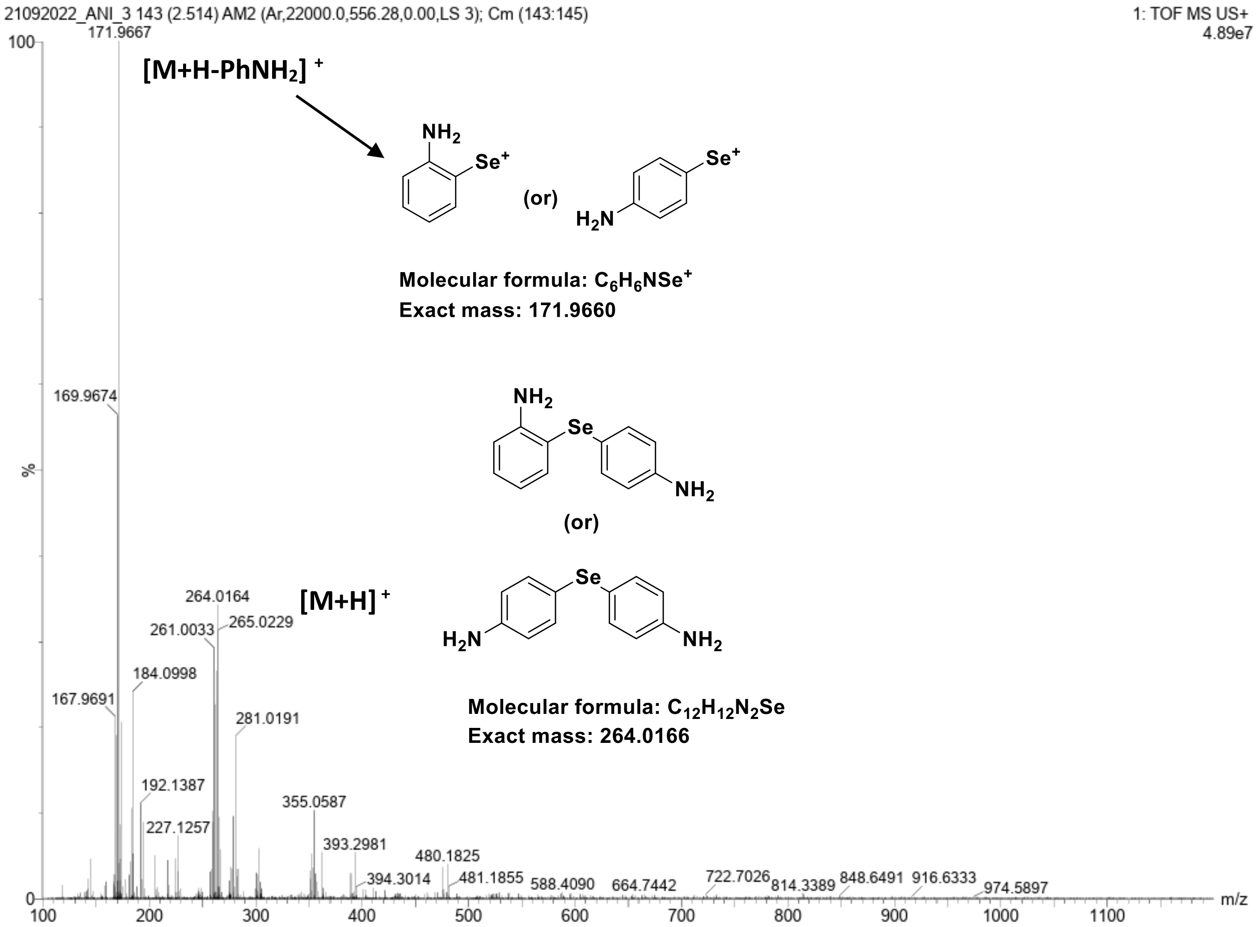

Figure S4. HRMS–ESI spectrum for mixture of compounds **1** and **2**.

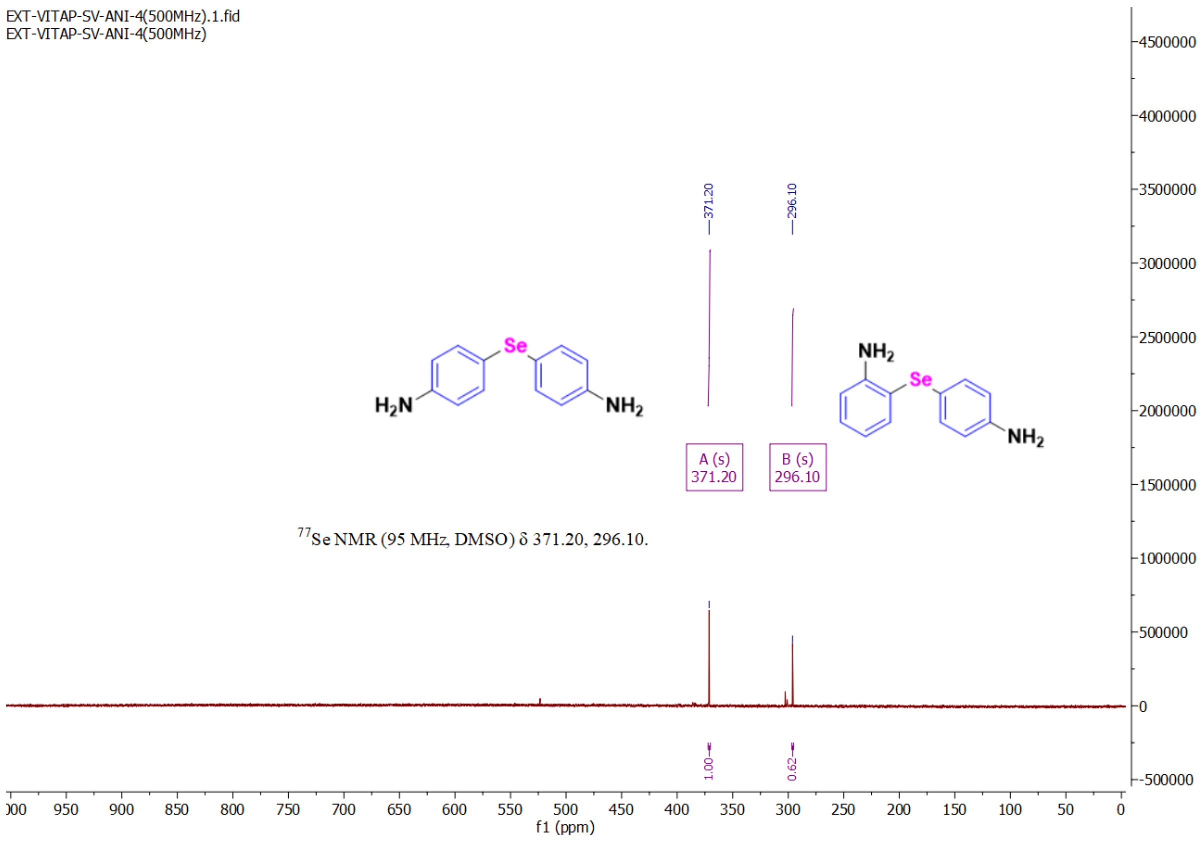

Figure S5. ^{77}Se NMR spectral studies for mixture of compounds **1** and **2**.

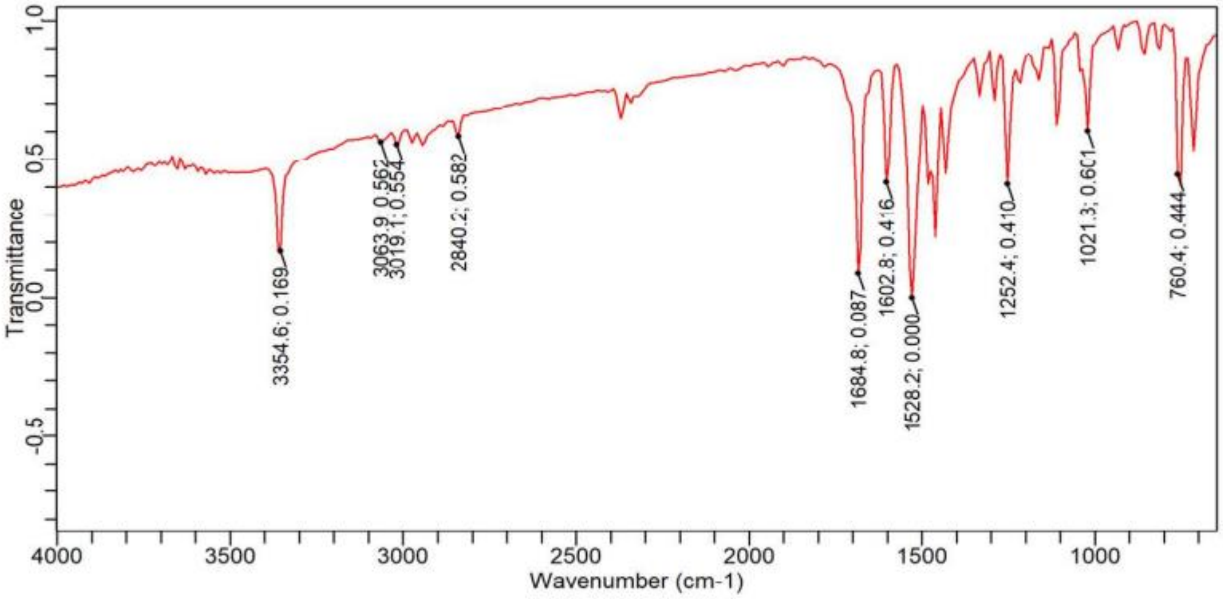

Figure S6. FTIR (KBr) spectrum for compound **3**.

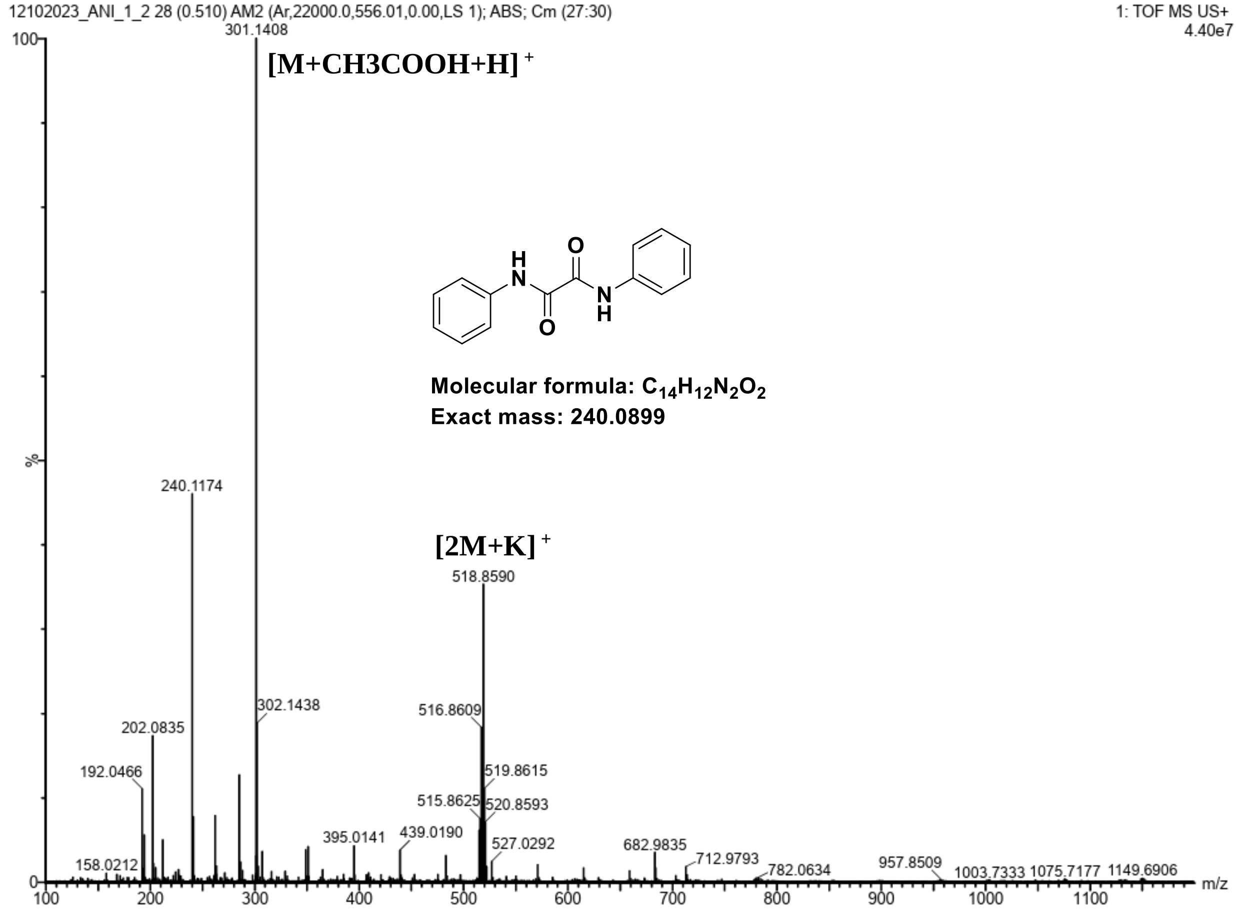

Figure S7. HRMS–ESI spectrum for compound **3**.

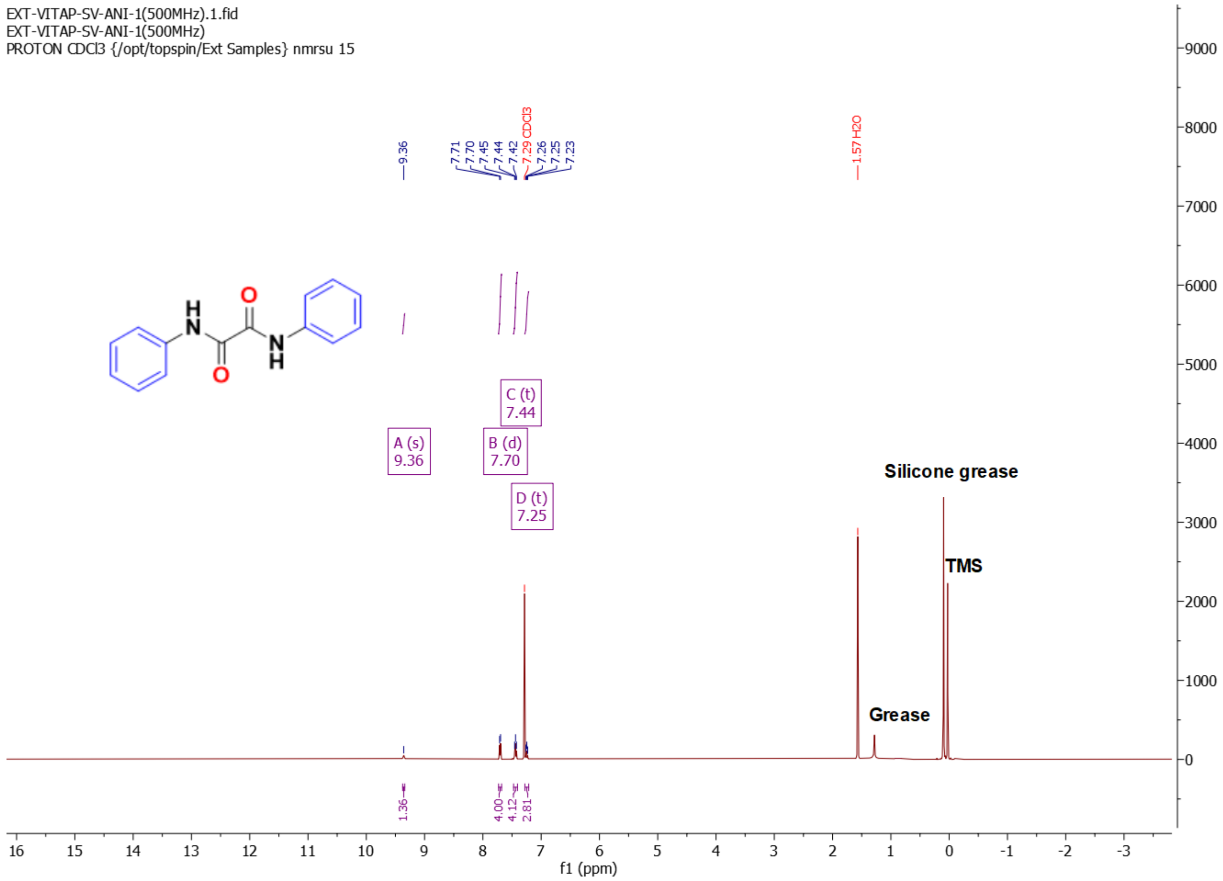

Figure S8. ^{1}H NMR spectral studies for compound **3**.

3. Spectrometric characterization of products obtained from the reaction of *o*-anisidine with SeO_2

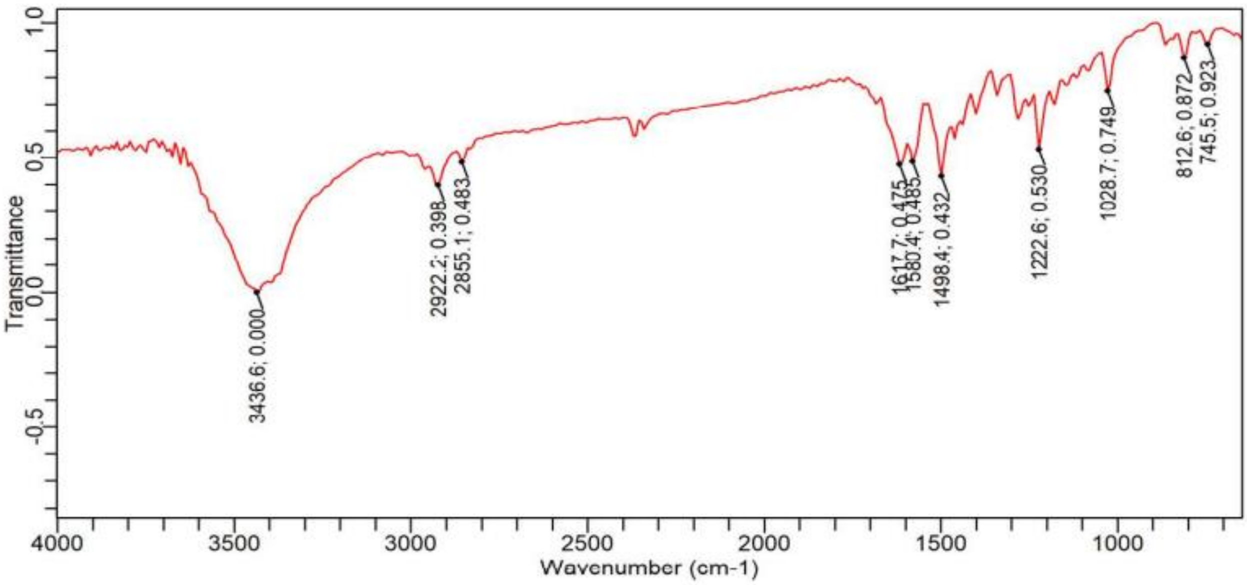

Figure S9. FTIR(KBr) spectrum for mixture of compounds **4**–**8**.

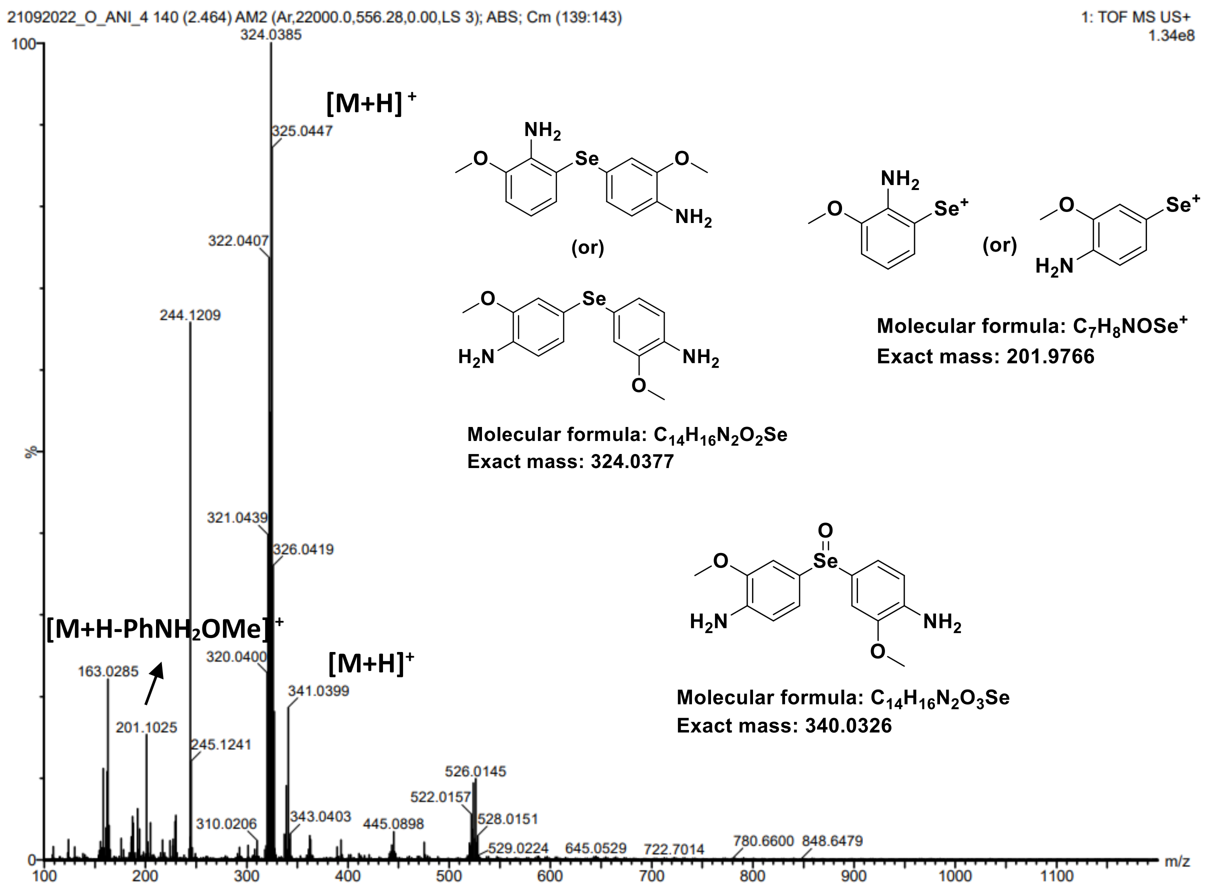

Figure S10. HRMS–ESI spectrum for mixture of compounds **4**–**8**.

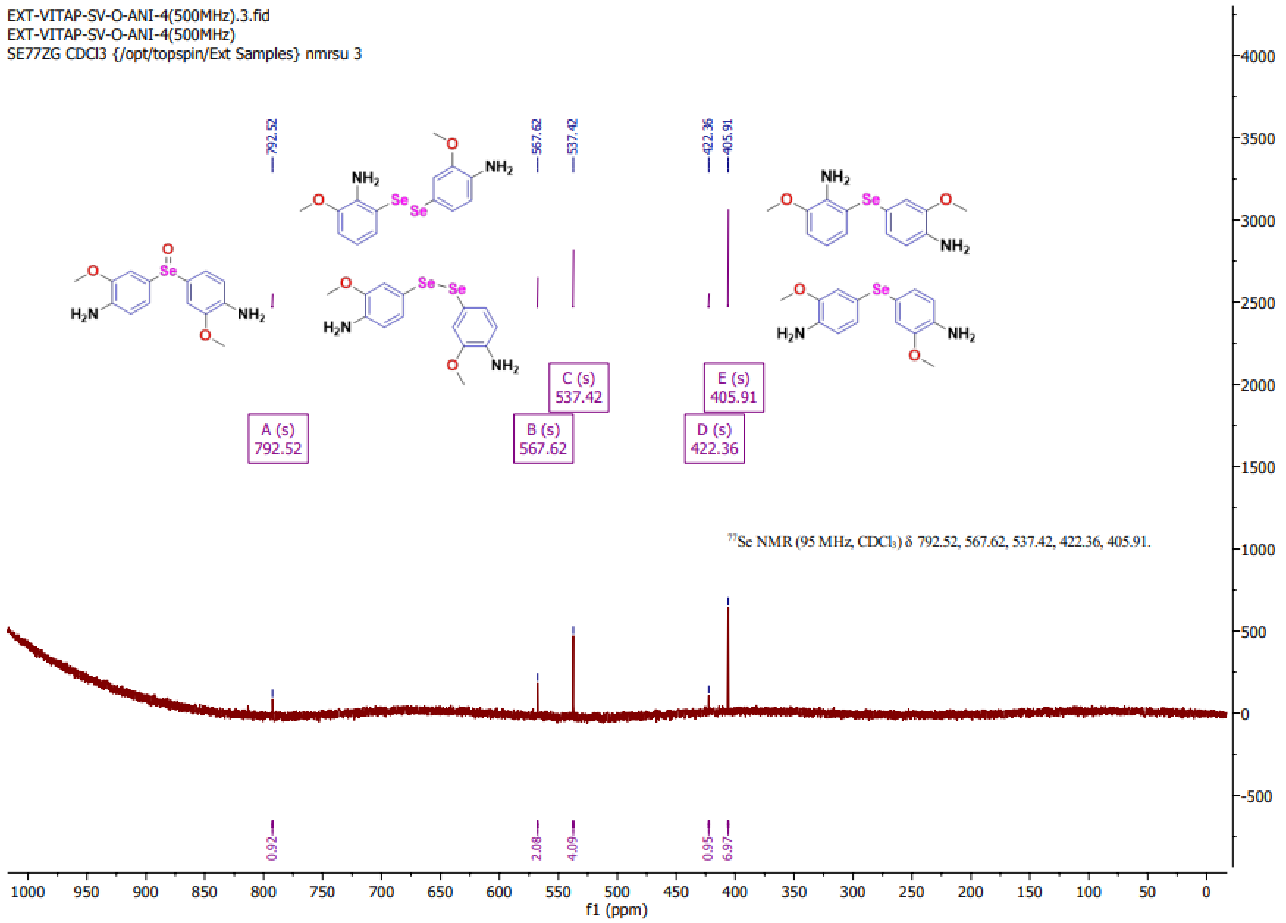

Figure S11. ^{77}Se NMR spectral studies for mixture of compounds **4**–**8**.

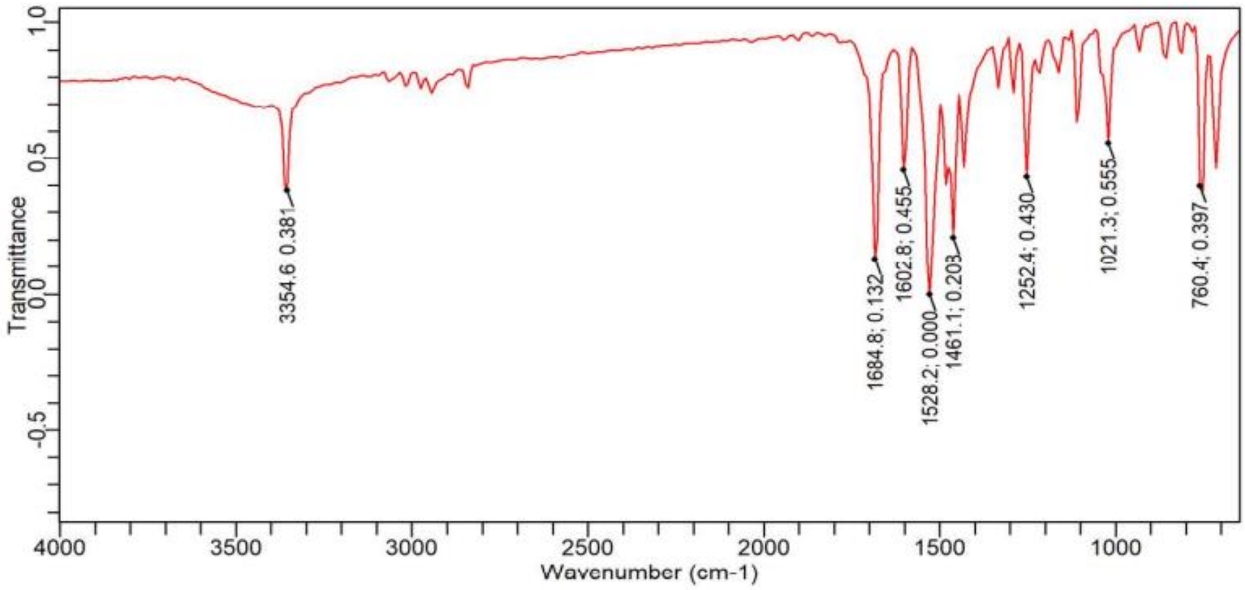

Figure S12. FTIR(KBr) spectrum for compound **9**.

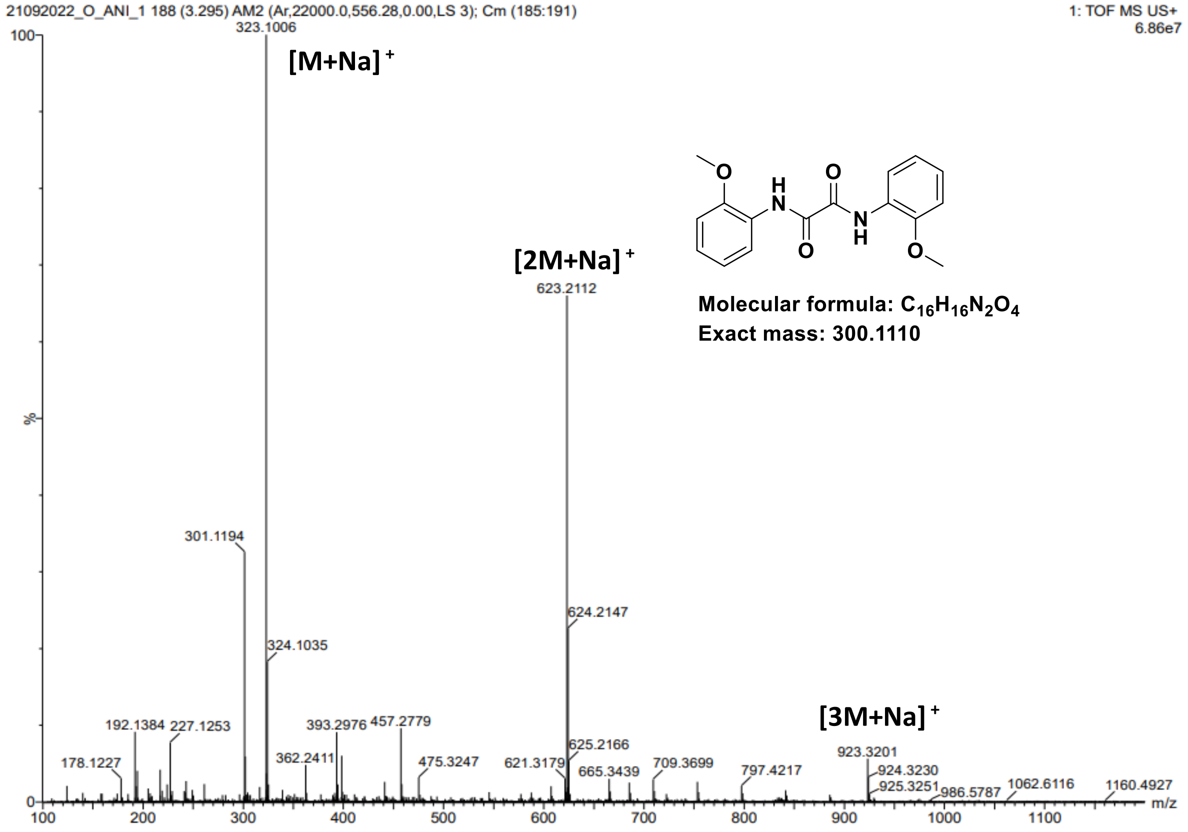

Figure S13. HRMS–ESI spectrum for compound **9**.

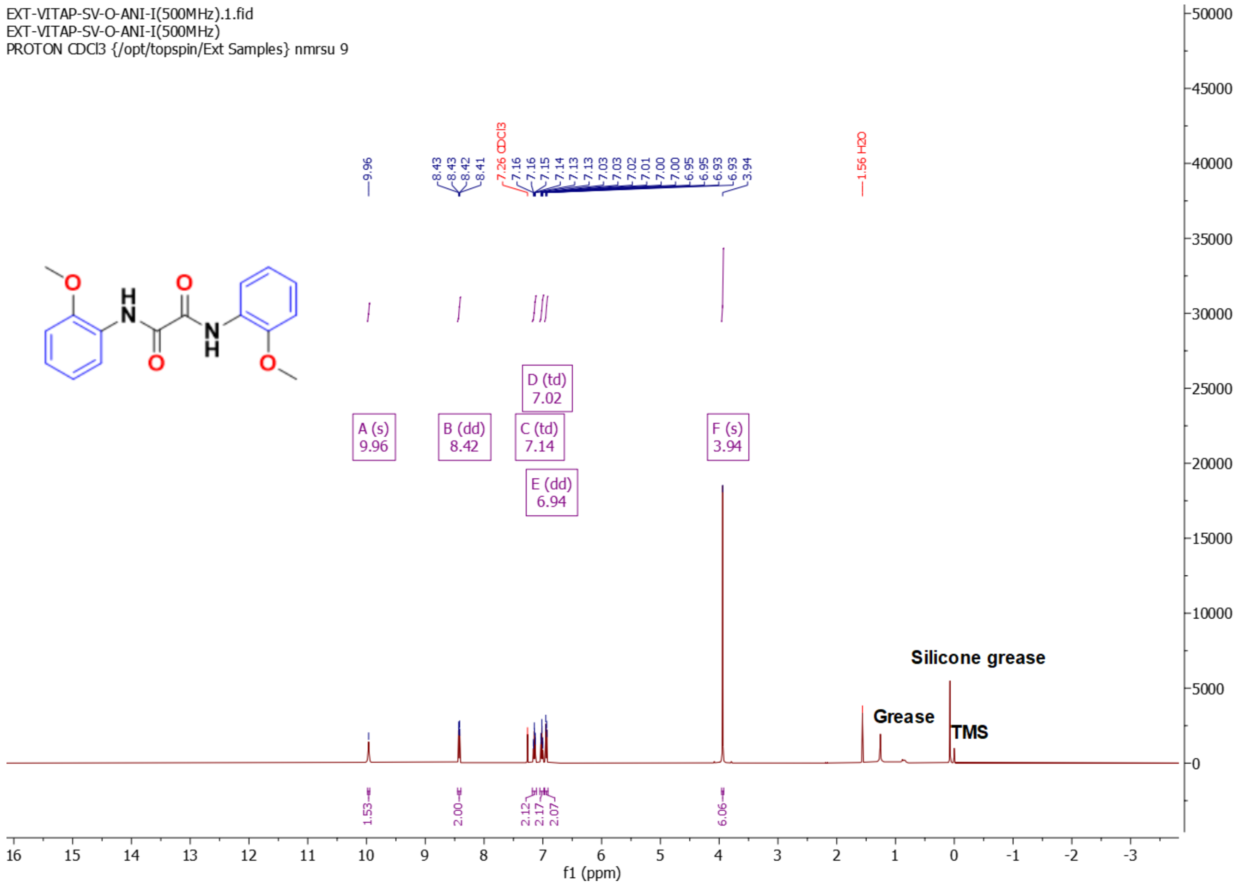

Figure S14. ^{1}H NMR spectral studies for compound **9**.

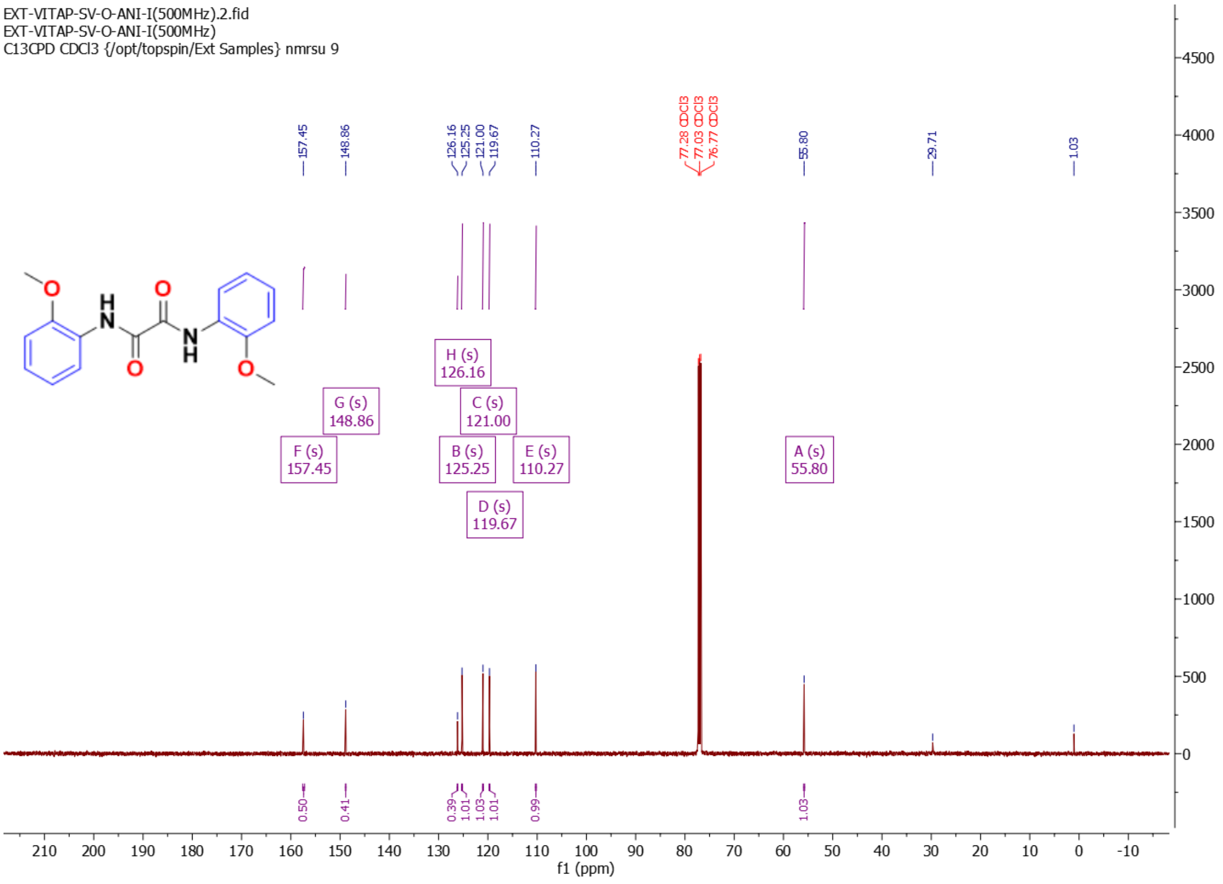

Figure S15. ^{13}C NMR spectral studies for compound **9**.

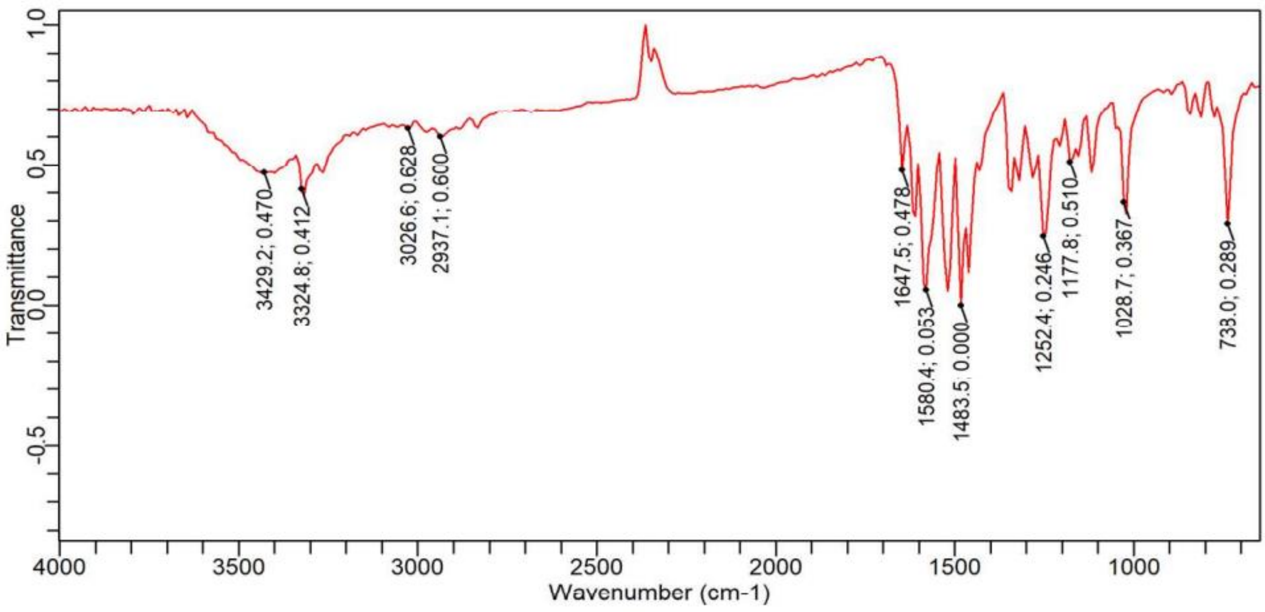

Figure S16. FTIR(KBr) spectrum for compound **10**.

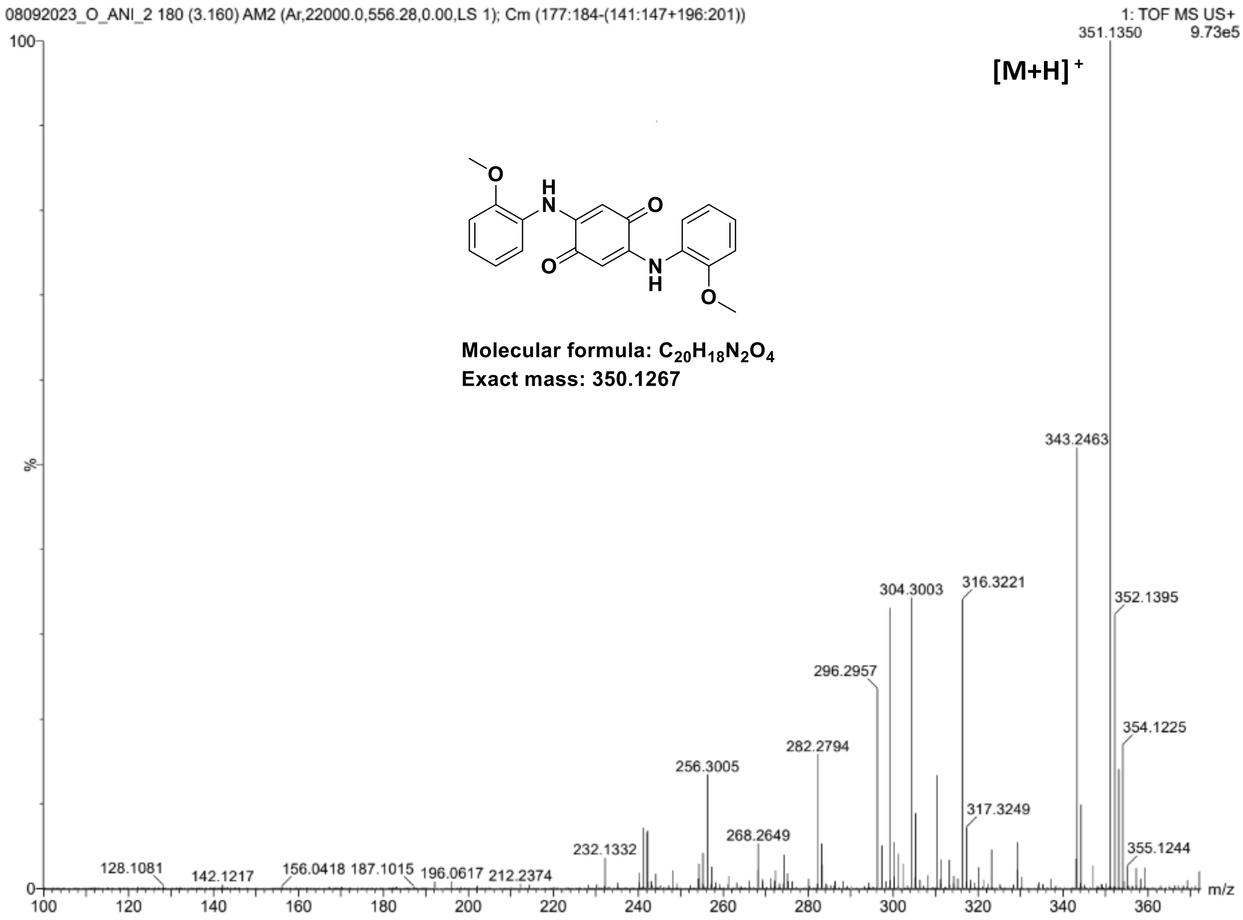

Figure S17. HRMS–ESI spectrum for compound **10**.

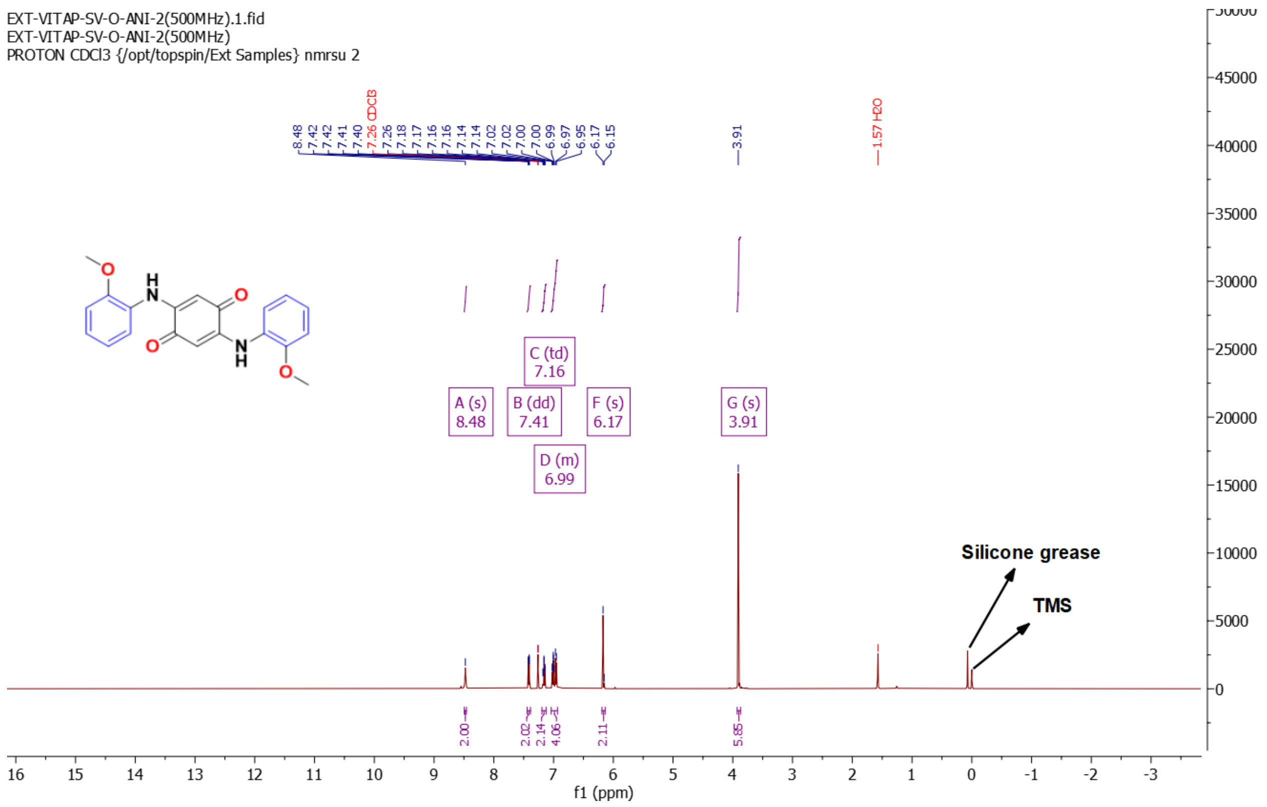

Figure S18. ^{1}H NMR spectral studies for compound **10**,

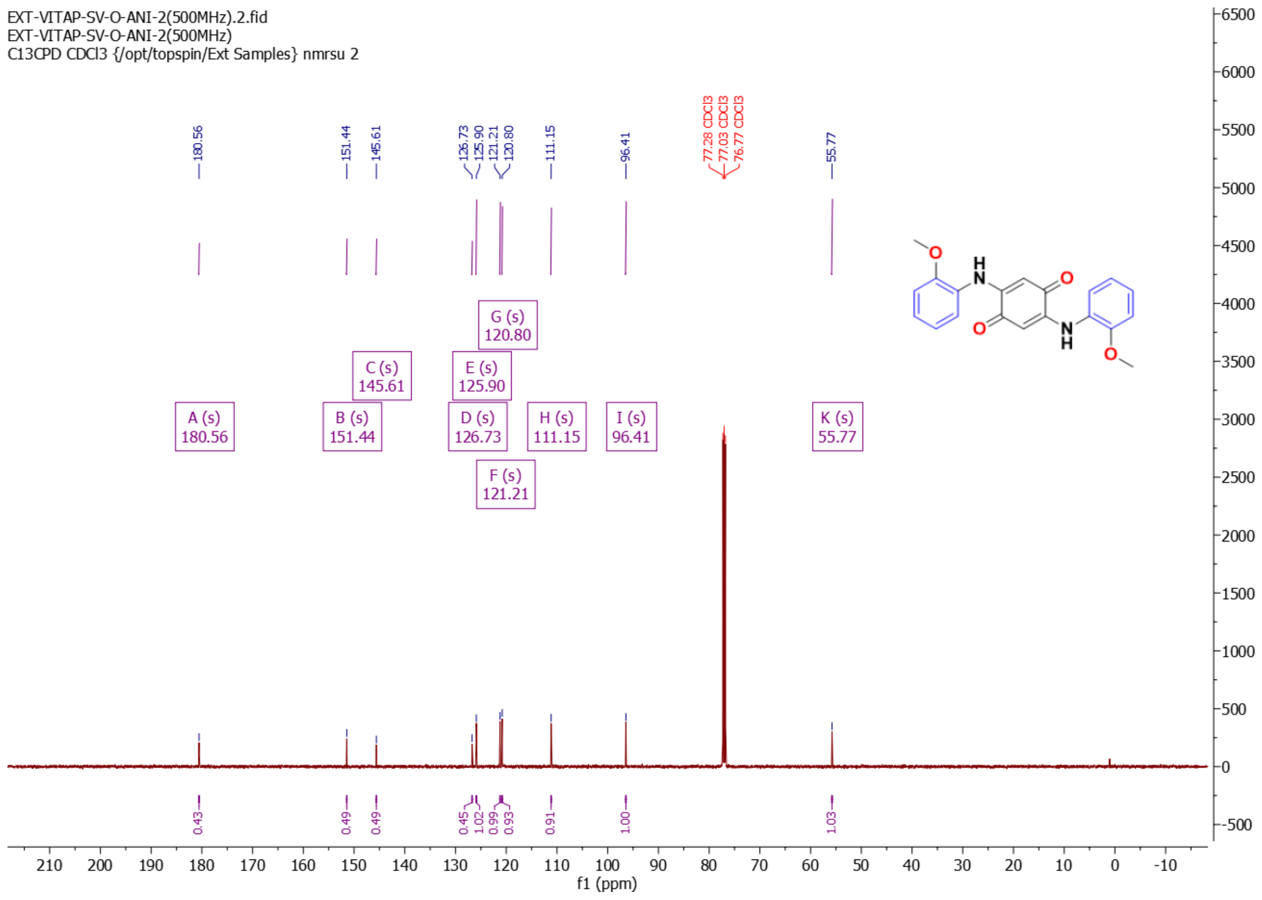

Figure S19. ^{13}C NMR spectral studies for compound **10**.

4. Spectrometric characterization of products obtained from the reaction of methyl anthranilate with SeO_2

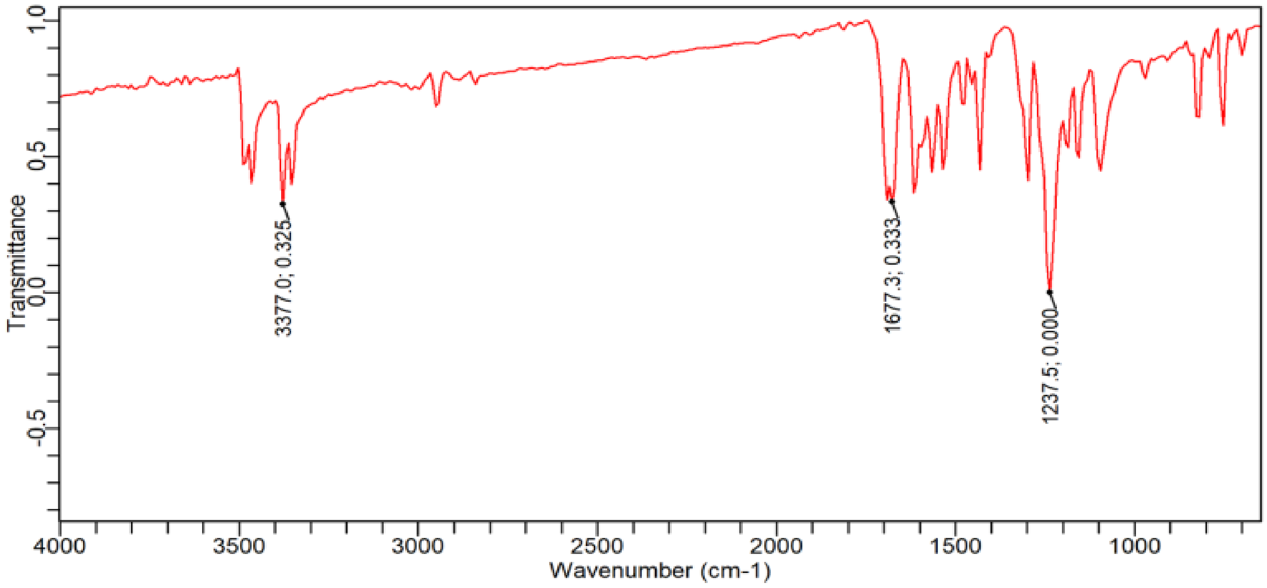

Figure S20. FTIR(KBr) spectrum for compound **11**.

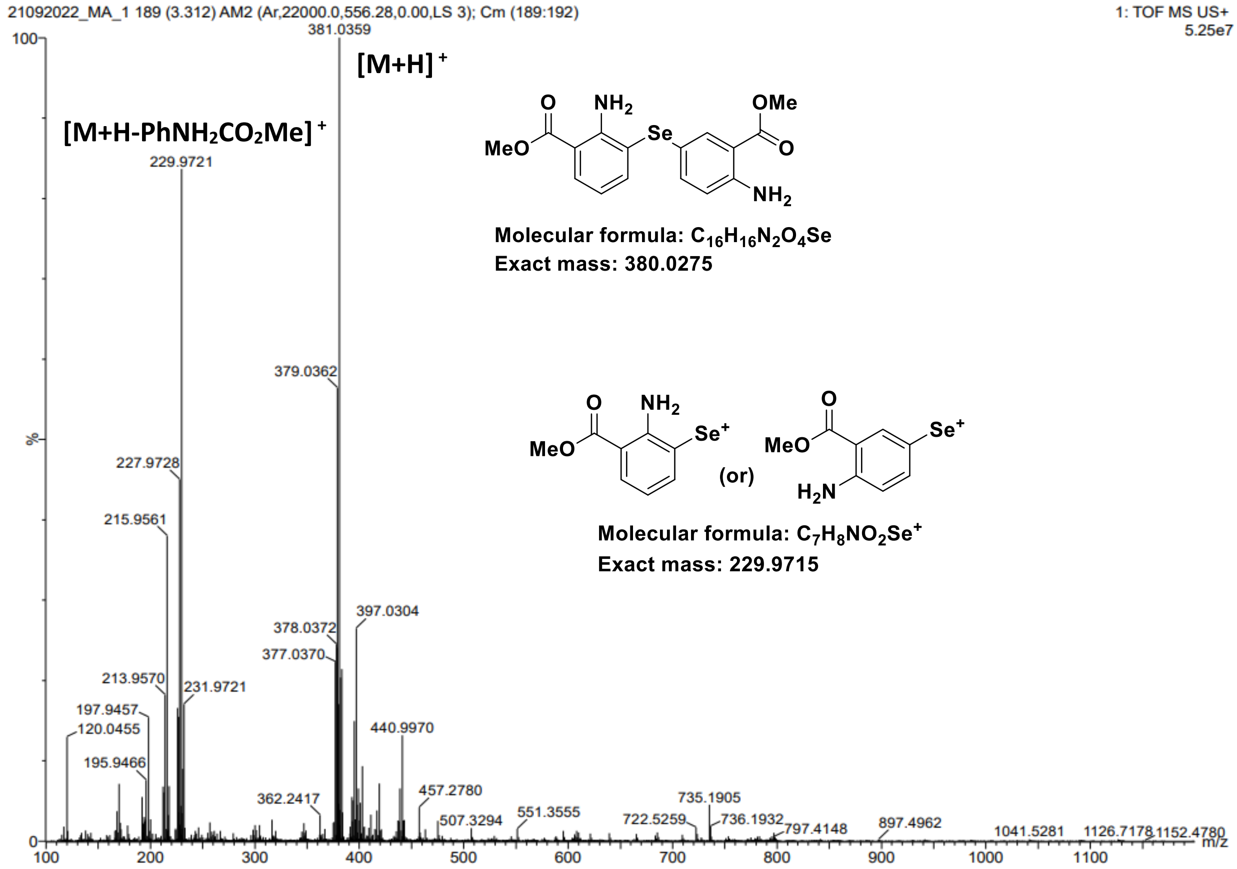

Figure S21. HRMS–ESI spectrum for compound **11**.

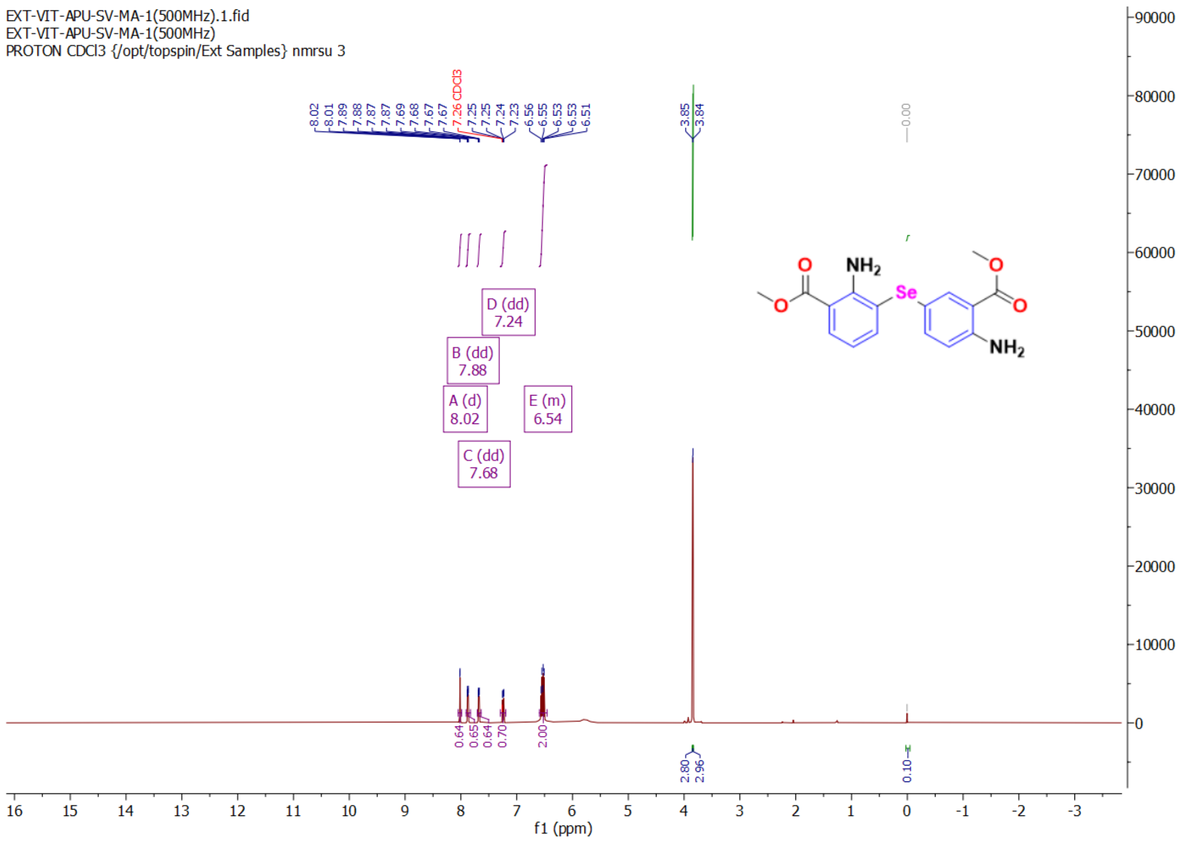

^{1}H NMR (500 MHz, $CDCl_3$) δ 8.02 (d, J = 2.2 Hz, 1H), 7.88 (dd, J = 8.1, 1.6 Hz, 1H), 7.68 (dd, J = 7.5, 1.7 Hz, 1H), 7.24 (dd, J = 8.6, 2.1 Hz, 1H), 6.59 – 6.45 (m, 3H), 3.84 (d, J = 3.1 Hz, 6H).

Figure S22. ^{1}H NMR spectral studies for compound **11**.

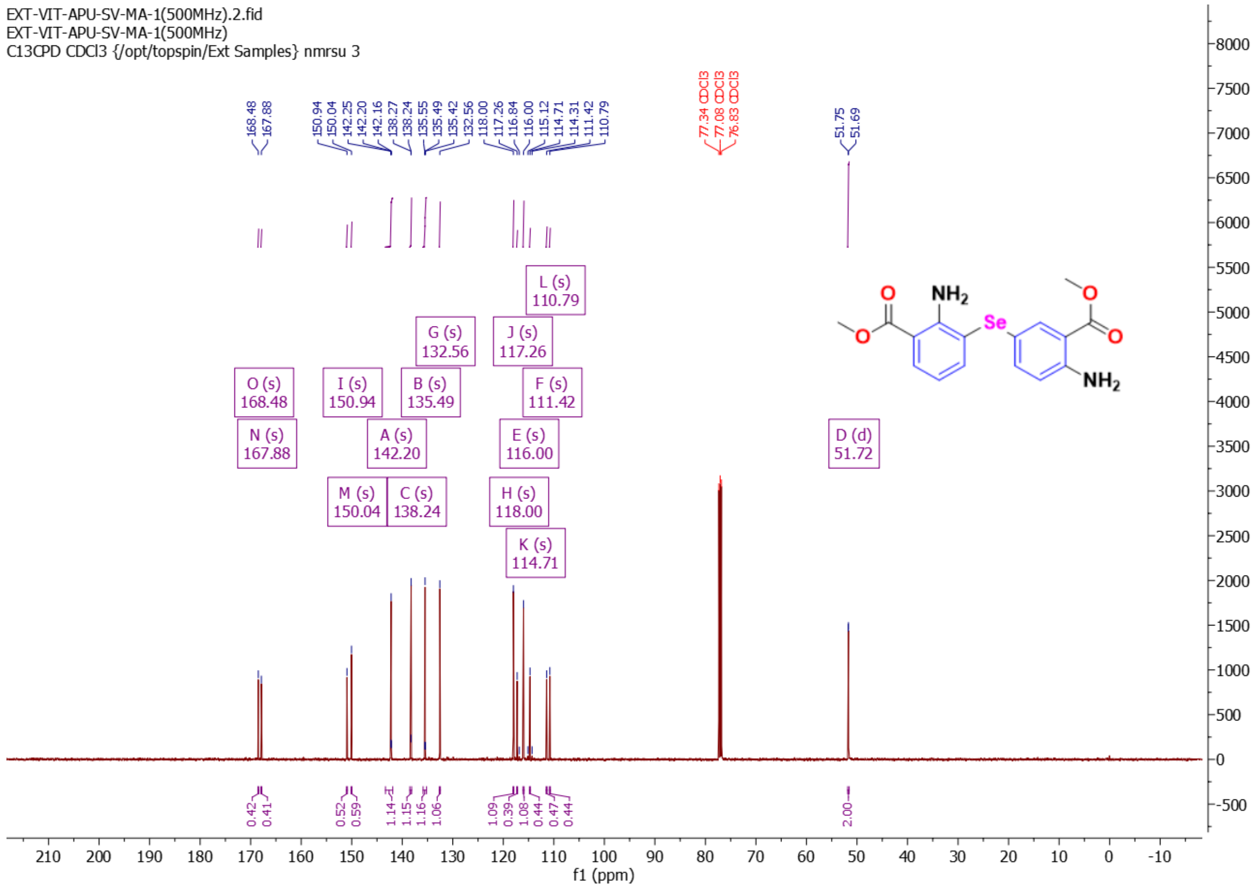

Figure S23. ^{13}C NMR spectral studies for compound **11**.

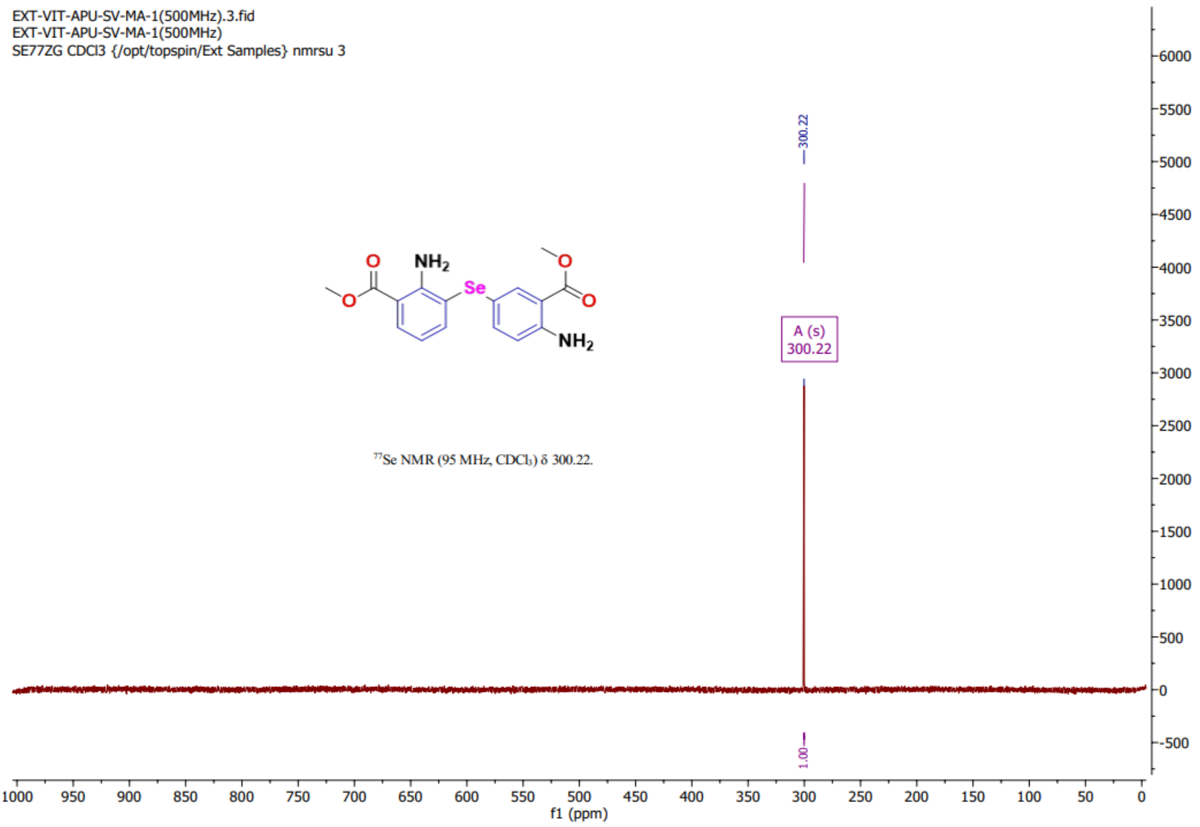

Figure S24. ^{77}Se NMR spectral studies for compound **11**.

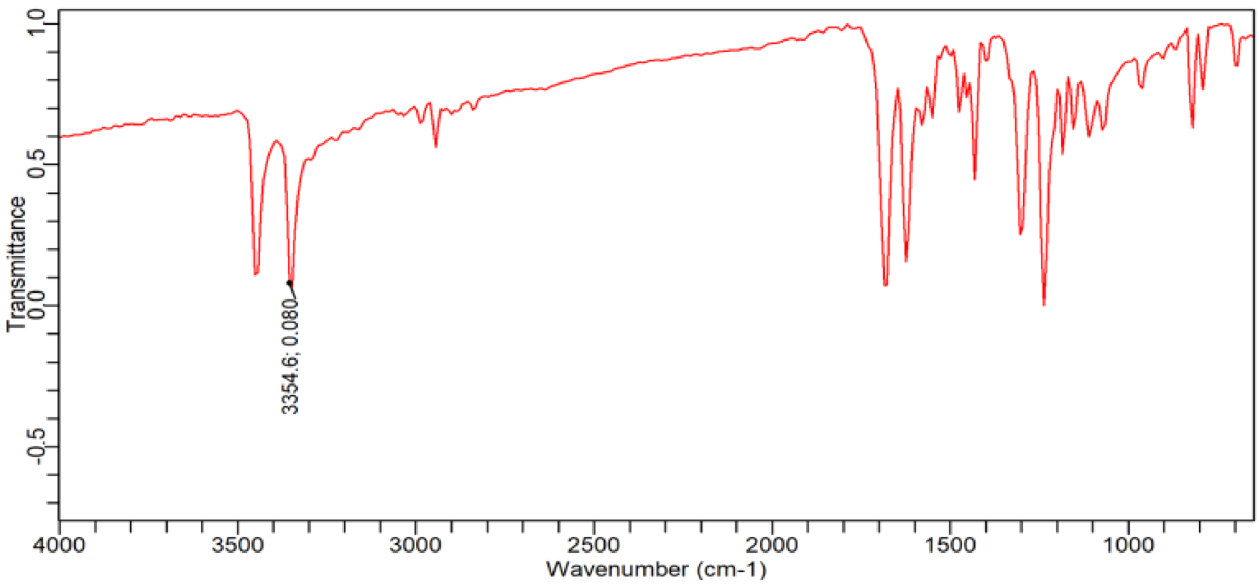

Figure S25. FTIR(KBr) spectrum for compound **12**.

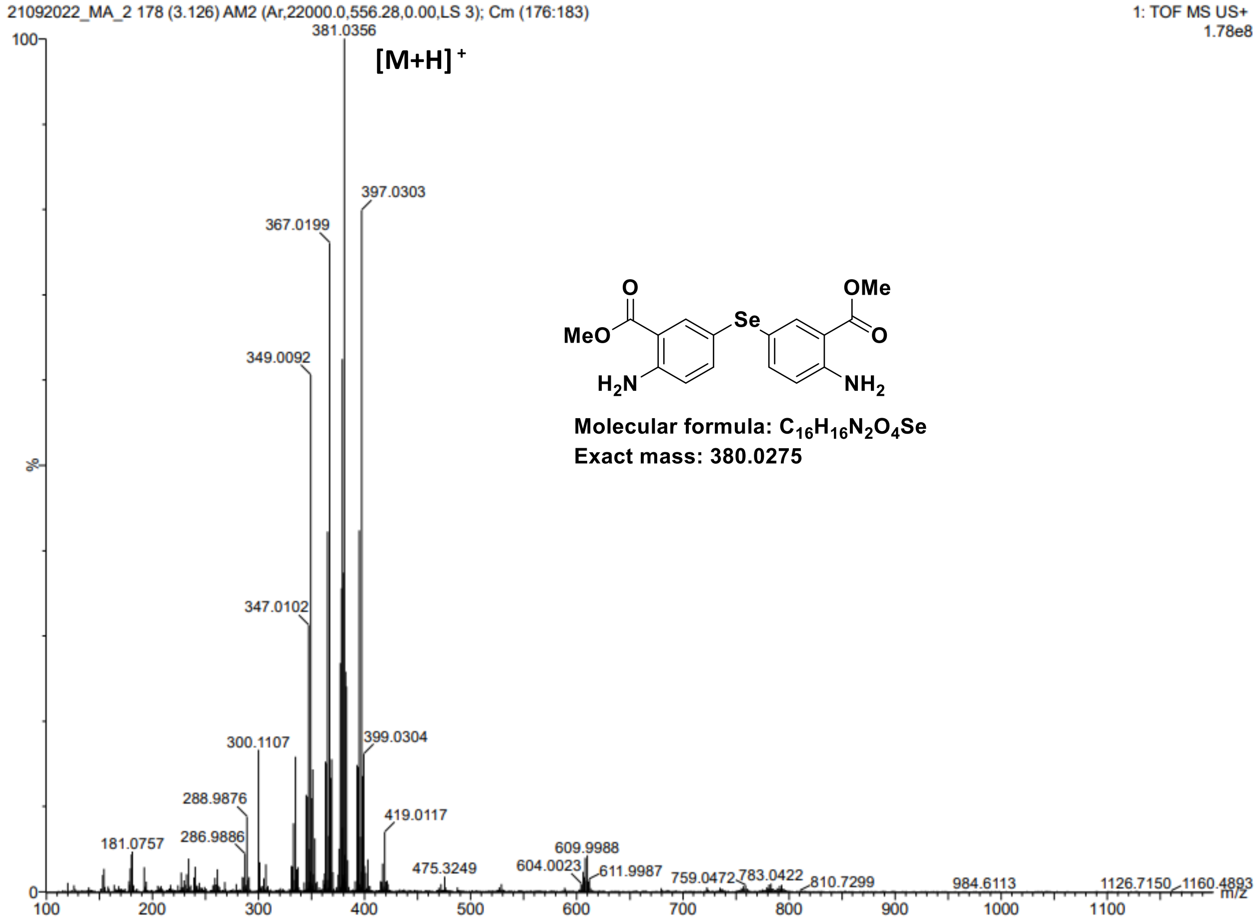

Figure S26. HRMS–ESI spectrum for compound **12**.

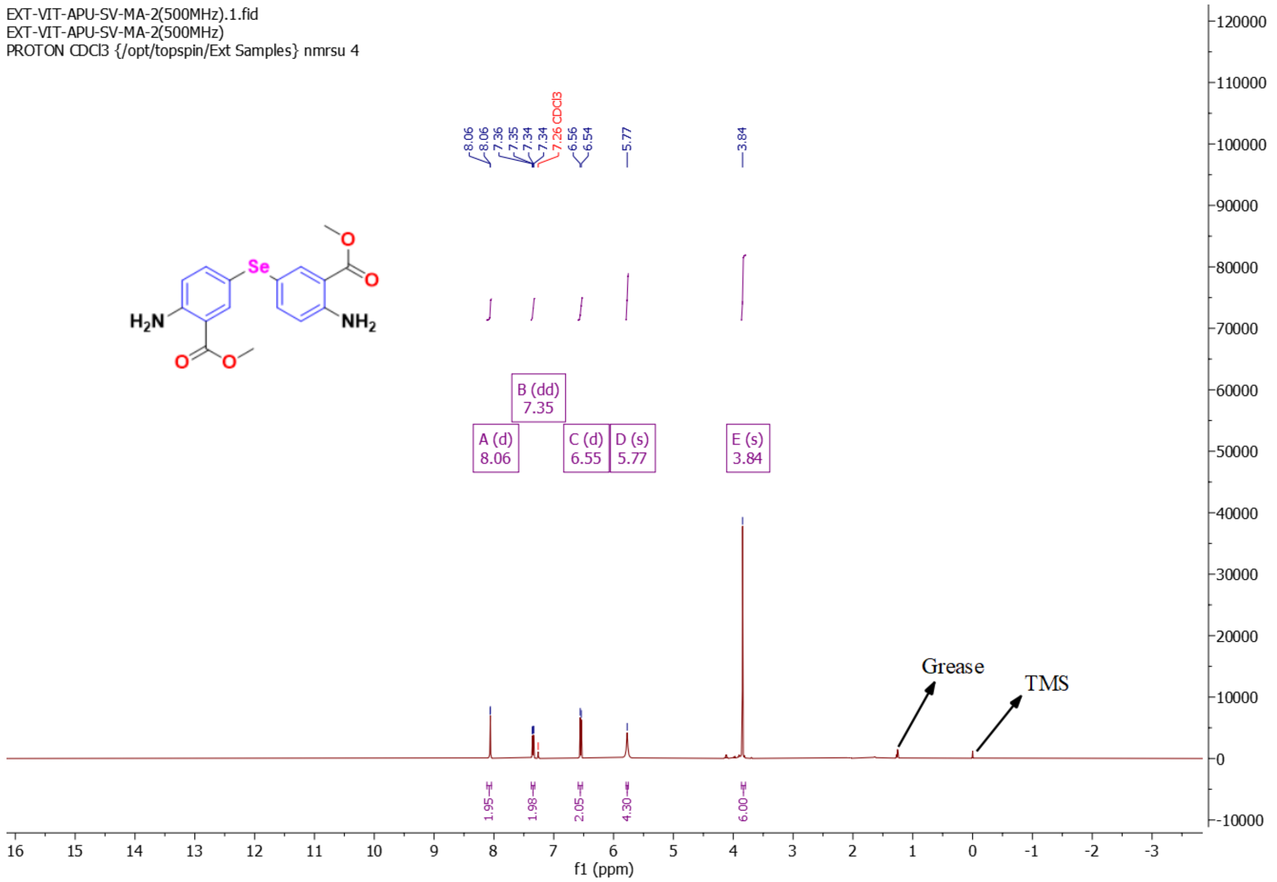

^{1}H NMR (500 MHz, $CDCl_3$) δ 8.06 (d, J = 2.1 Hz, 1H), 7.35 (dd, J = 8.6, 2.1 Hz, 1H), 6.55 (d, J = 8.5 Hz, 1H), 5.77 (s, 2H), 3.84 (s, 3H).

Figure S27. ^{1}H NMR spectral studies for compound **12**.

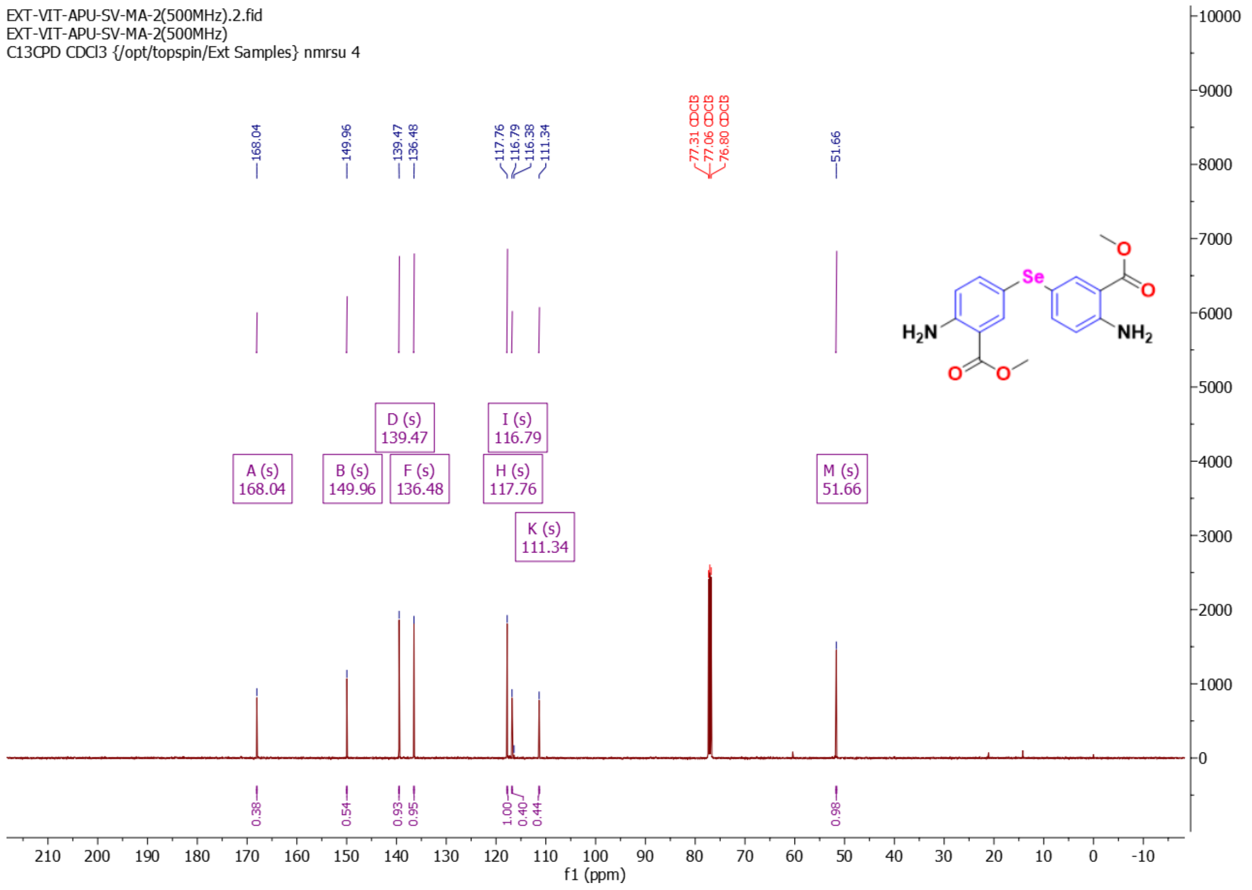

Figure S28. ^{13}C NMR spectral studies for compound **12**.

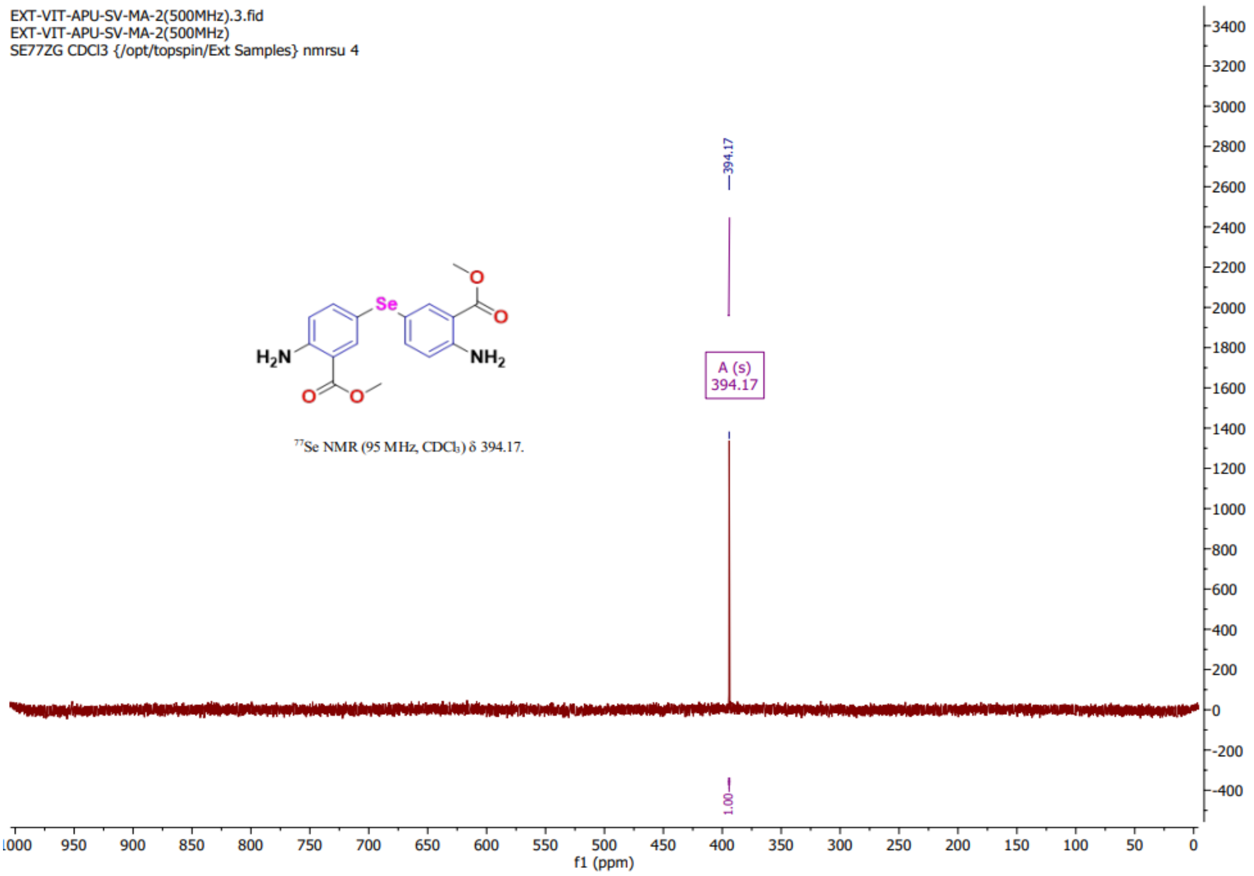

Figure S29. ^{77}Se NMR spectral studies for compound **12**.

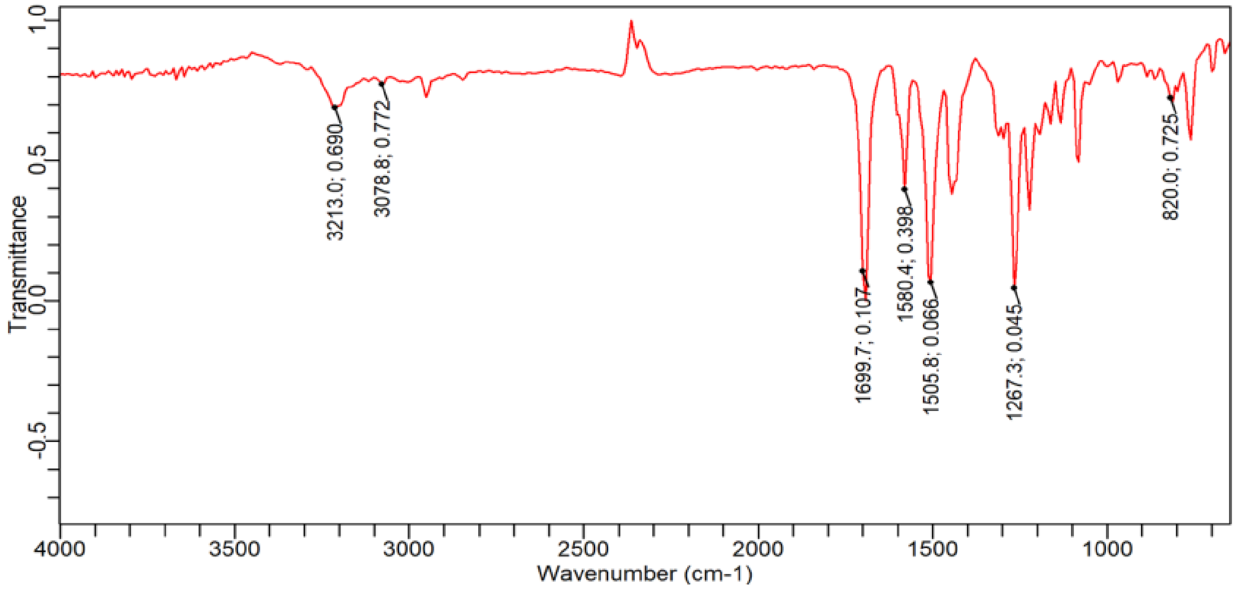

Figure S30. FTIR(KBr) spectrum for compound **13**.

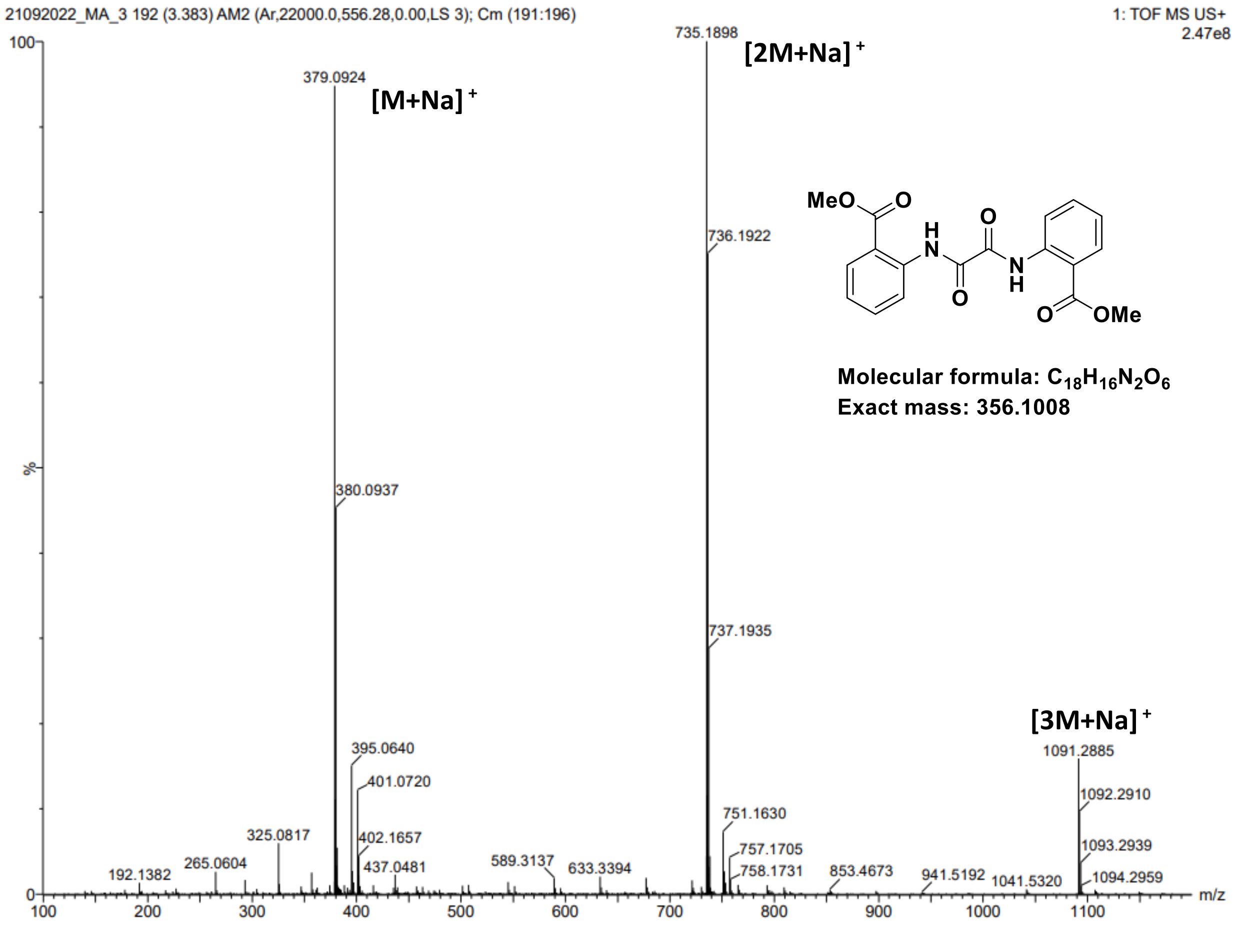

Figure S31. HRMS–ESI spectrum for compound **13**.

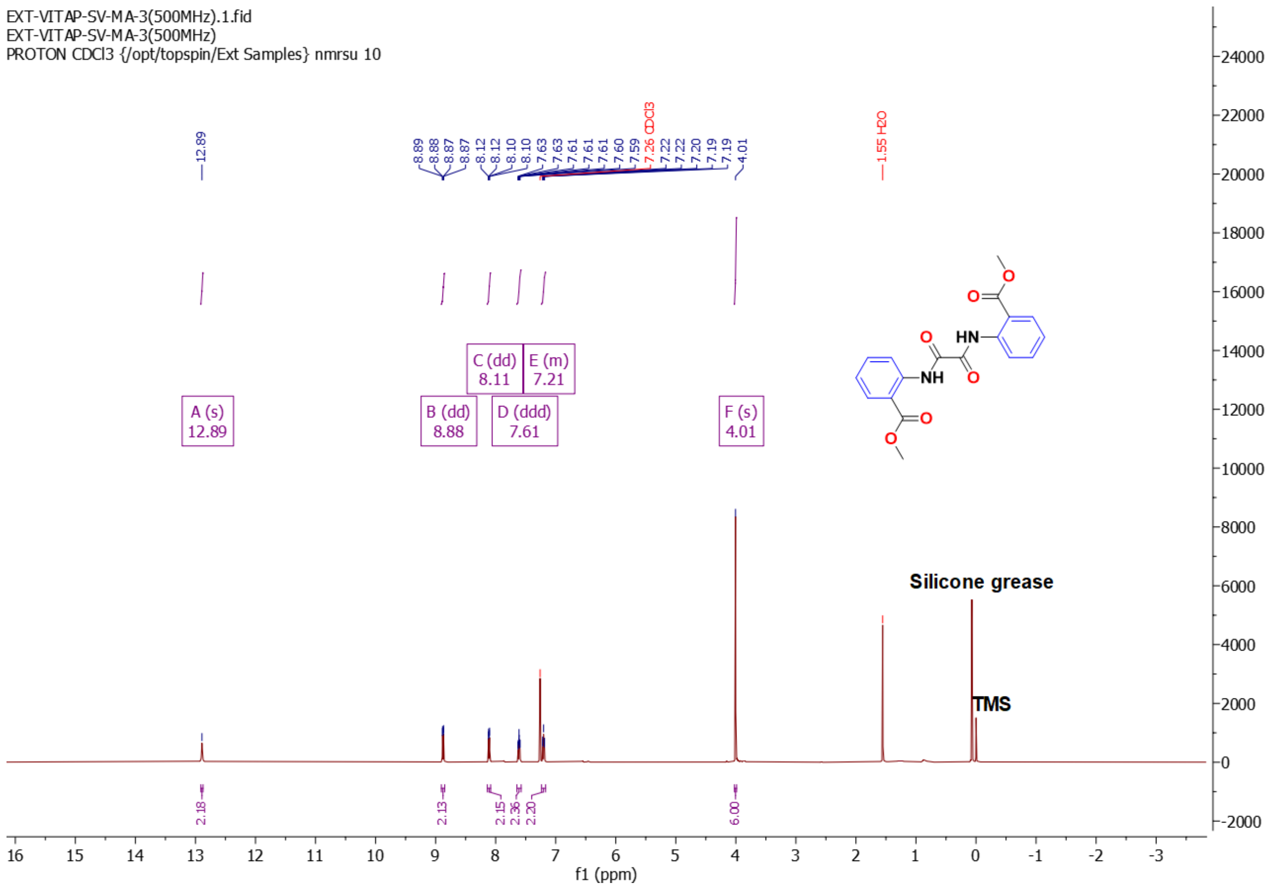

Figure S32. ^{1}H NMR spectral studies for compound **13**.

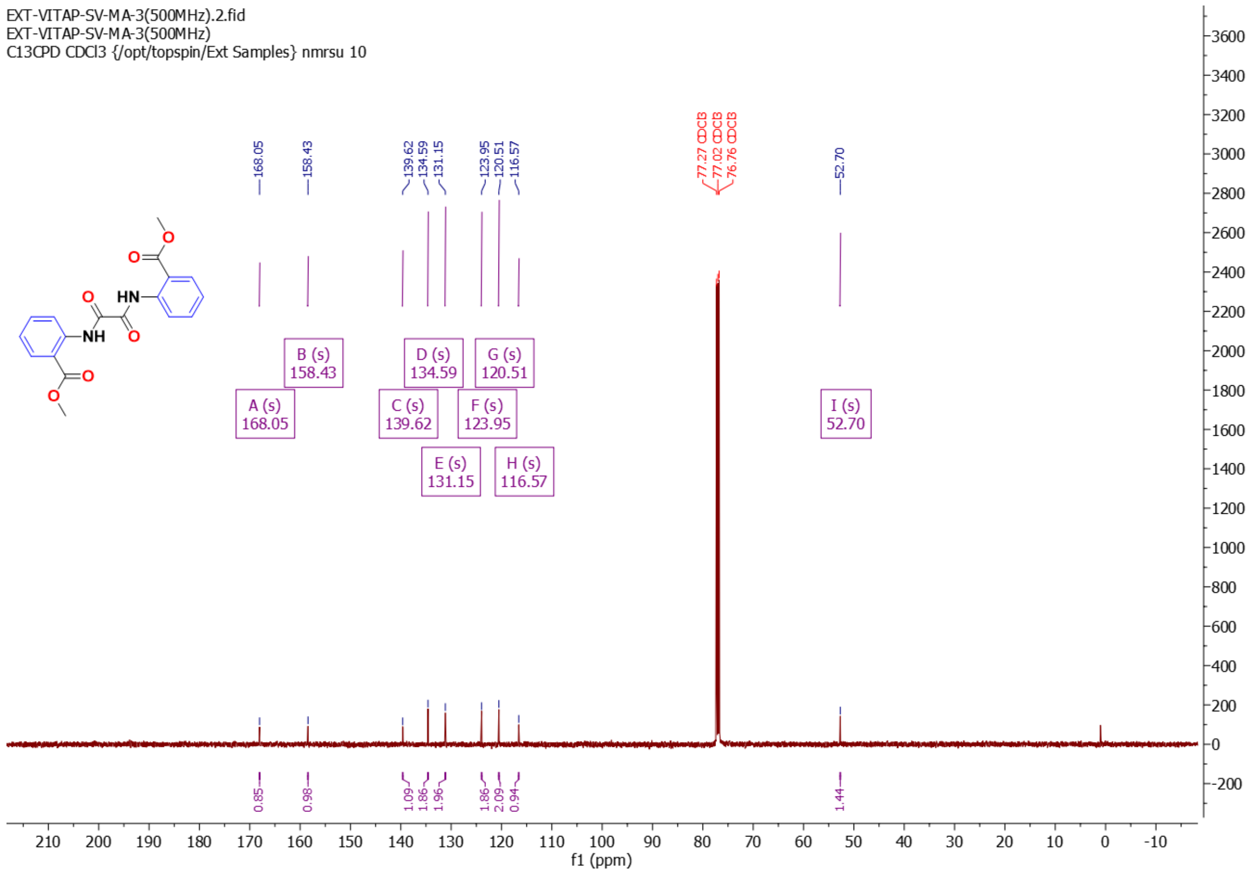

Figure S33. ^{13}C NMR spectral studies for compound **13**.

5. Relative color change upon addition of SeO_2 to arylamines in acetonitrile

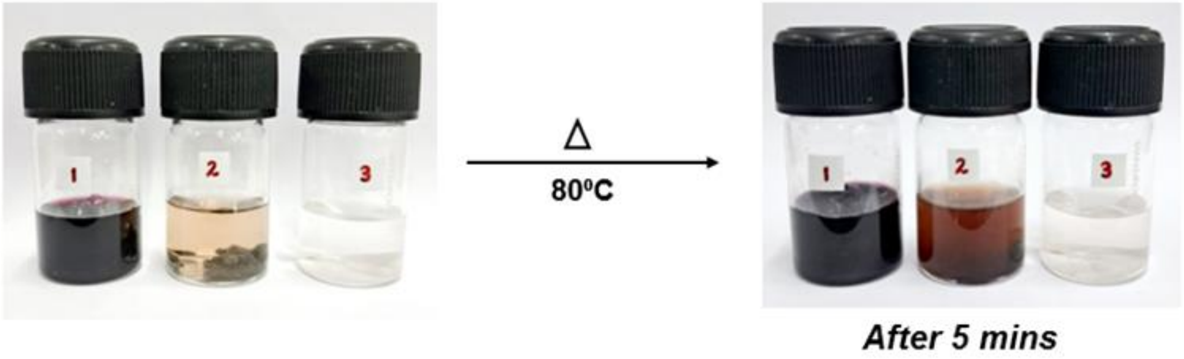

Figure S34: Relative color change upon addition of SeO_2 to arylamines in acetonitrile: vial 1: *o*-anisidine, vial 2: aniline, vial 3: methyl anthranilate.

6. Packing arrangement for compounds 3 and 9

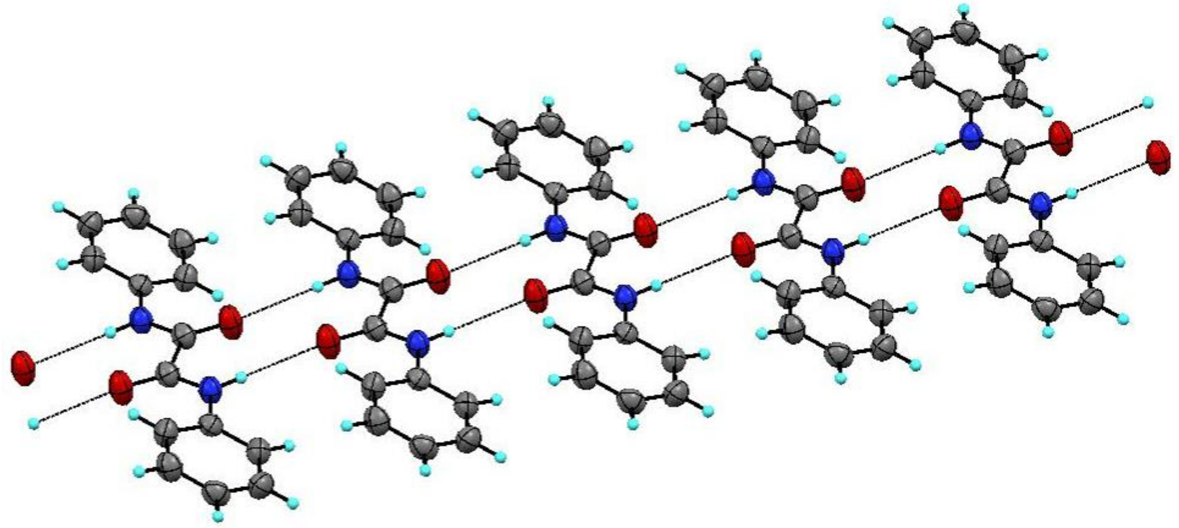

Figure S35. Packing arrangement for compound **3**.

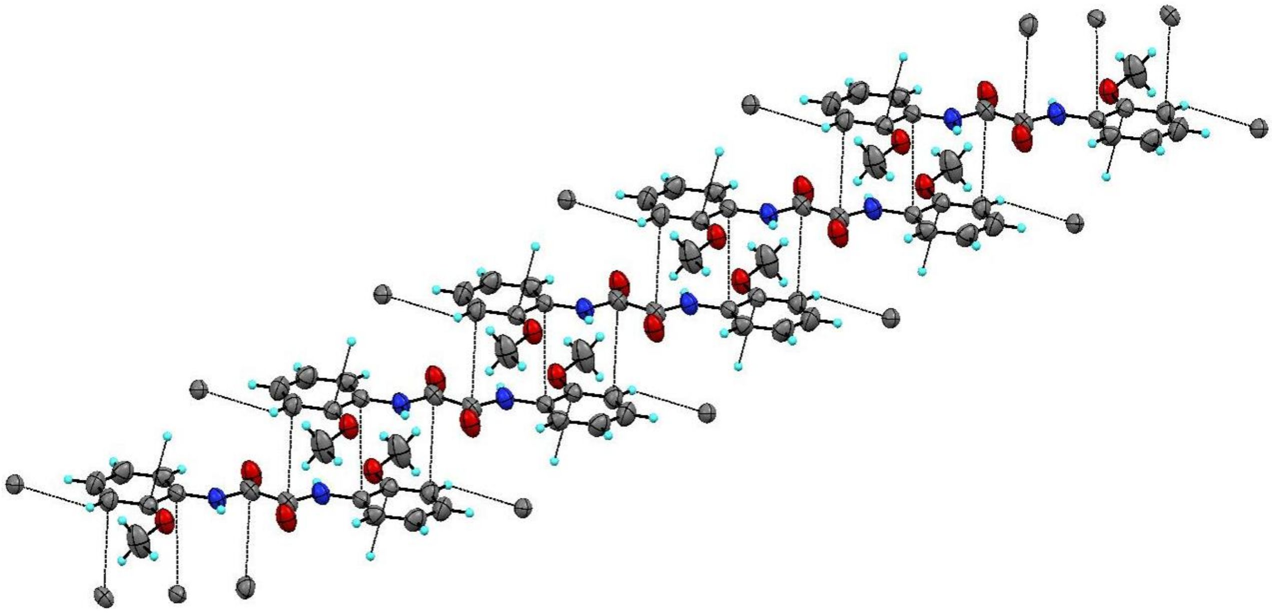

Figure S36. Packing arrangement for compound **9**.

7. Packing arrangement for compounds 13 and 10

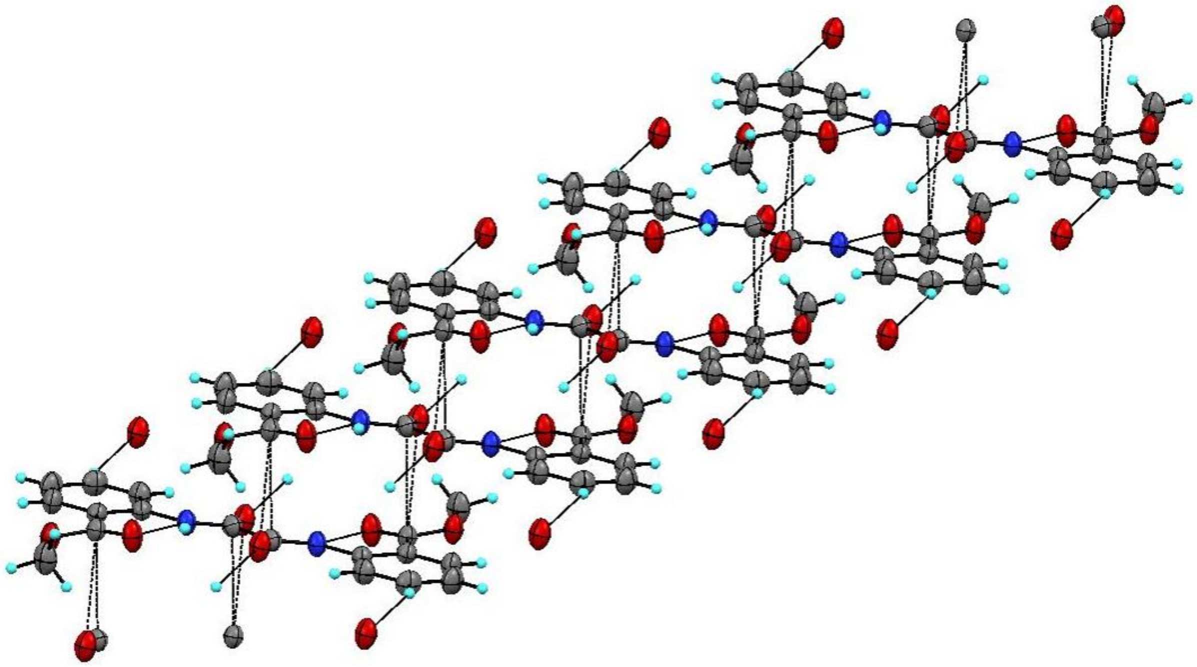

Figure S37. Packing arrangement for compound **13**.

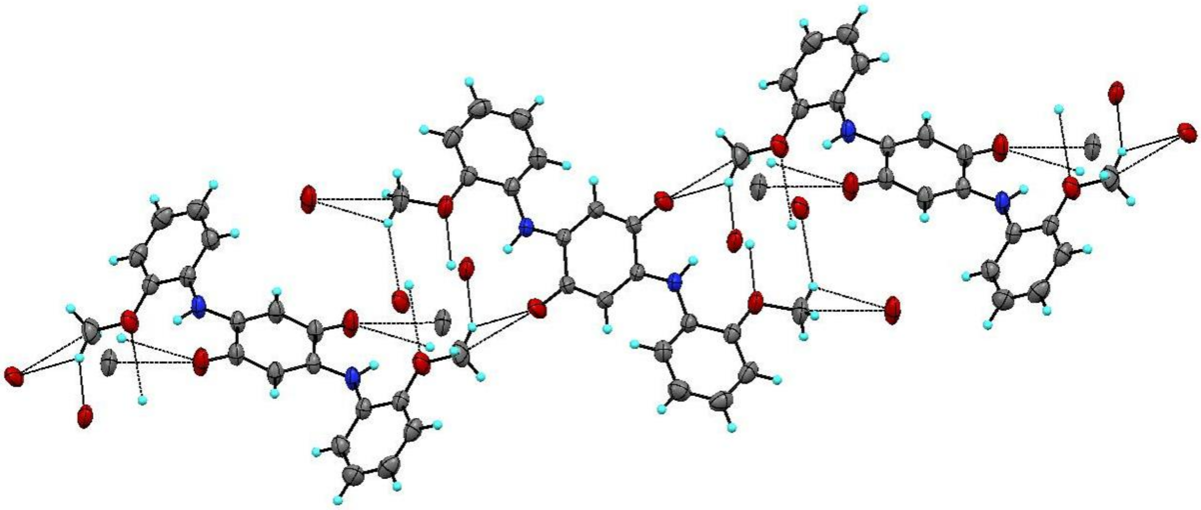

Figure S38. Packing arrangement for compound **10**.

8. Packing arrangement for compound 11

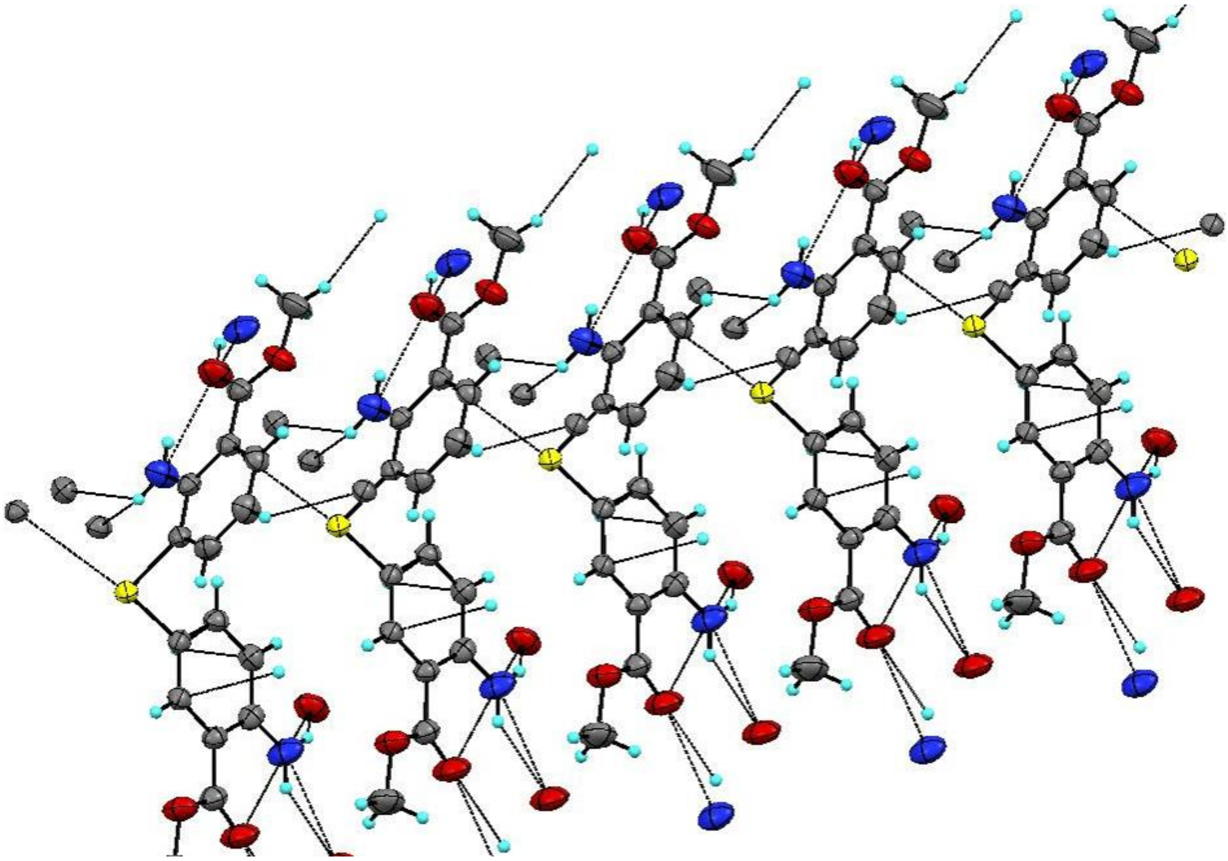

Figure S39. Packing arrangement for compound **11**.

9. Packing arrangement for compound 12

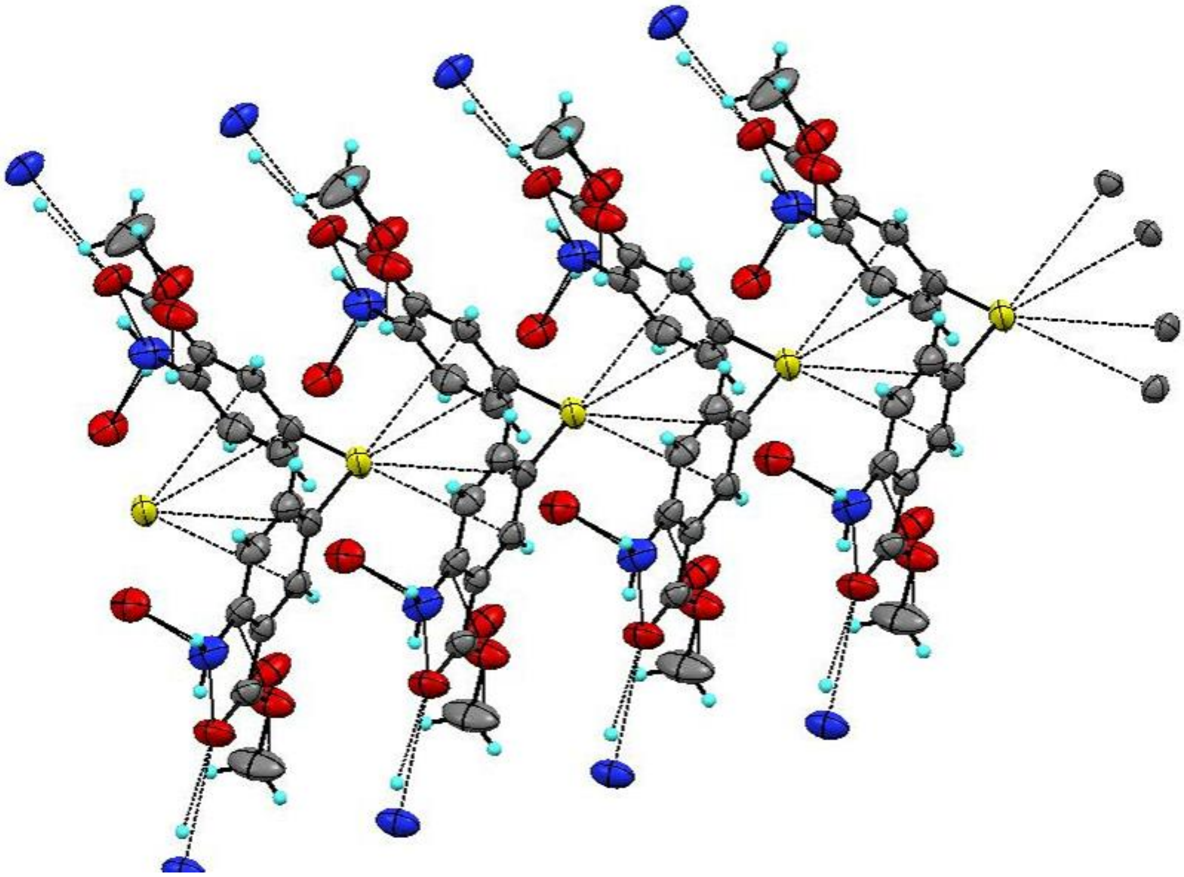

Figure S40. Packing arrangement for compound **12**.

10. HOMO and LUMO energy values for reactants

reactant	HOMO (donor)<br>kJ/mol	LUMO (acceptor)<br>kJ/mol
o–anisidine	-517.9324<br>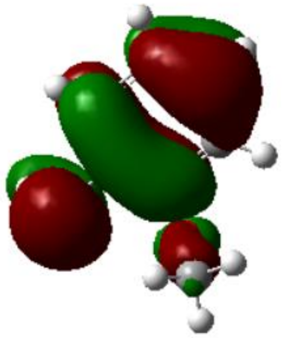	24.2334<br>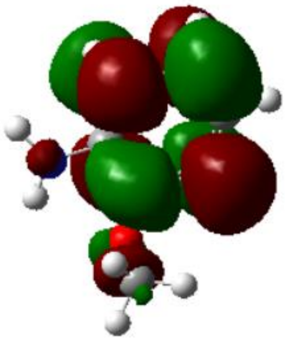
aniline	-520.4003<br>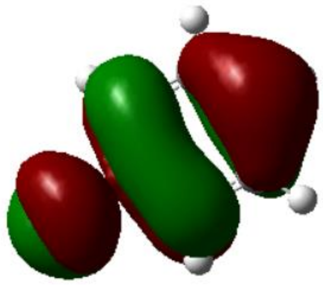	22.3955<br>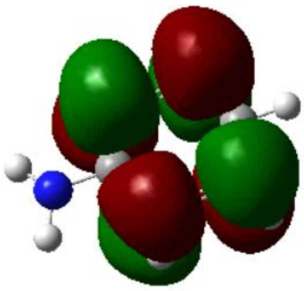
methyl anthranilate	-526.2552<br>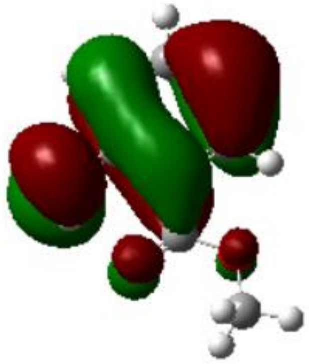	-91.9450<br>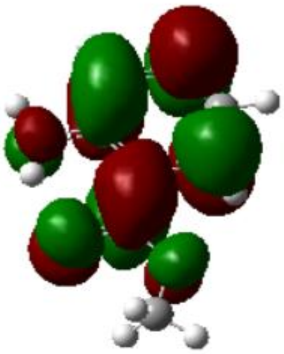

Selenium dioxide	-820.8101 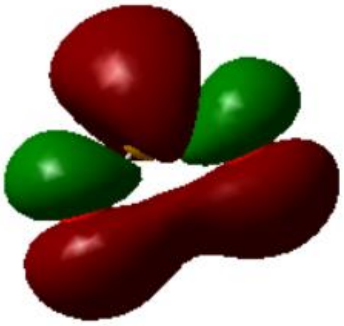	-394.4289 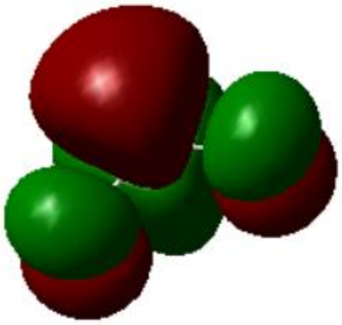

Figure S41: HOMO and LUMO energy values for reactants.
